# Signatures of a magnetic superstructure phase induced by ultrahigh magnetic fields in a breathing pyrochlore antiferromagnet

**DOI:** 10.1073/pnas.2302756120

**Published:** 2023-08-07

**Authors:** Masaki Gen, Akihiko Ikeda, Kazushi Aoyama, Harald O. Jeschke, Yuto Ishii, Hajime Ishikawa, Takeshi Yajima, Yoshihiko Okamoto, Xuguang Zhou, Daisuke Nakamura, Shojiro Takeyama, Koichi Kindo, Yasuhiro H. Matsuda, Yoshimitsu Kohama

**Affiliations:** ^a^Institute for Solid State Physics, University of Tokyo, Kashiwa, Chiba 277-8581, Japan; ^b^RIKEN Center for Emergent Matter Science, Wako, Saitama 351-0198, Japan; ^c^Department of Engineering Science, University of Electro-Communications, Chofu, Tokyo 182-8585, Japan; ^d^Department of Earth and Space Science, Graduate School of Science, Osaka University, Osaka 560-0043, Japan; ^e^Research Institute for Interdisciplinary Science, Okayama University, Okayama 700-8530, Japan

**Keywords:** frustrated magnetism, breathing anisotropy, spin–lattice coupling, ultrahigh magnetic fields

## Abstract

The mutual coupling of spin and lattice degrees of freedom is ubiquitous in magnetic materials and potentially creates exotic magnetic states in response to the external magnetic field. Particularly, geometrically frustrated magnets serve as a fertile playground for realizing magnetic superstructure phases. Here, we observe an unconventional two-step magnetostructural transition prior to a half-magnetization plateau in a breathing pyrochlore chromium spinel by means of state-of-the-art magnetization and magnetostriction measurements in ultrahigh magnetic fields available up to 600 T. Considering a microscopic magnetoelastic theory, the intermediate-field phase can be assigned to a magnetic superstructure with a three-dimensional periodic array of 3-up-1-down and canted 2-up-2-down spin molecules. We attribute the emergence of the magnetic superstructure to a unique combination of the strong spin–lattice coupling and large breathing anisotropy.

## Significance

The search for exotic magnetic states, such as a quantum spin liquid and a magnetic superstructure, in geometrically frustrated magnets has been a central research topic in the recent 30 y. Theoretically, the mutual coupling of spin and lattice degrees of freedom has been proposed to induce rich magnetic phases in various spin models. Here, we observe an unconventional multistep magnetostructural transition in a breathing pyrochlore antiferromagnet in ultrahigh magnetic fields, signaling the emergence of a magnetic superstructure phase with a periodic array of collinear 3-up-1-down and canted antiferromagnetic states. This finding could be attributed to the interplay between the spin–lattice coupling and breathing anisotropy.

Superstructures in crystalline solids, where the unit cell of atomic arrangements or electronic states is an integer multiple of the original primitive cell of the lattice, have received considerable attention because of their complexity as well as their association with exotic physical properties. Prominent examples are surface superstructures such as the Si(111)–7 × 7 state (1), charge/orbital ordering in perovskite manganites (2), and charge density wave states in van der Waals transition-metal dichalcogenides (3) and topological kagome superconductors (4). For frustrated spin systems, a variety of quantum-entangled magnetic superstructures can appear in the external magnetic field. Of particular interest are a series of magnon crystals in a spin-1/2 kagome Heisenberg antiferromagnet (5) and successive transformations of singlet-triplet superstructures in a spin-1/2 orthogonal-dimer Heisenberg antiferromagnet (6). These phenomena are accompanied by a cascade of fractional magnetization plateaus (7–11).

**Fig. 1.**
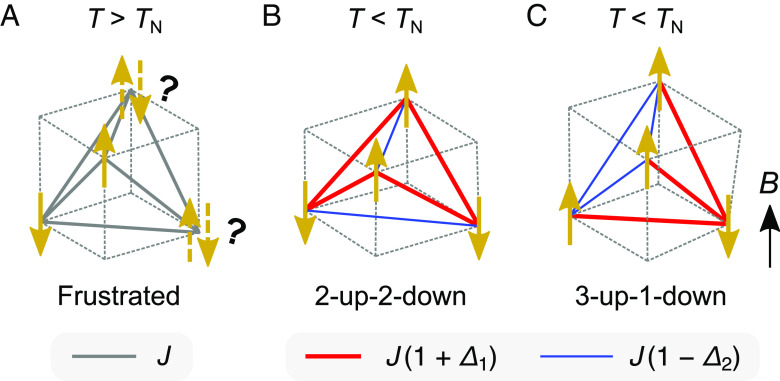
Spin configurations in a local tetrahedron of the classical Heisenberg antiferromagnet on a pyrochlore lattice. The magnetic frustration arising from the geometrical constraint (*A*) is relieved by the spin Jahn–Teller effect below the ordering temperature *T*_N_ in zero field, resulting in a 2-up-2-down state (*B*). On application of a magnetic field *B*, the system undergoes a phase transition to a 3-up-1-down state (*C*). In both collinear magnetic states, the AFM exchange interaction in contracted (elongated) bonds colored by red (blue) becomes stronger (weaker) by *J∆*_1_ (*J∆*_2_) than the original strength *J* through the lattice deformation (25).

When a spin system interacts with the lattice degrees of freedom, the structural instability can facilitate the formation of a magnetic long-range order, potentially yielding a spin–lattice-coupled superstructure. The concept of a spin–lattice coupling was first theoretically proposed as the spin-Peierls transition in a spin-1/2 Heisenberg antiferromagnetic (AFM) chain (12, 13), which was then demonstrated in a number of quasi-one-dimensional compounds (14, 15). In this mechanism, the spin dimerization is induced by individual atomic displacements with a doubling of the unit cell, where the system gains exchange energy at the cost of elastic energy. A similar phase transition was subsequently found in three-dimensional semiclassical spin systems represented by chromium-based spinels *A*Cr_2_O_4_ (*A* = Zn, Cd, and Hg) (16–19). At low temperatures, the strong geometrical frustration inherent in the corner-sharing tetrahedral network, i.e., the pyrochlore lattice, of spin-3/2 Cr^3+^ ions is relieved by the local tetrahedral distortion (called the spin Jahn–Teller effect), resulting in a 2-up-2-down magnetic long-range order (16, 19) (Fig. 1 *A* and *B*). Interestingly, the application of a magnetic field induces another spin–lattice-coupled long-range order, a 3-up-1-down state (19, 20) (Fig. 1*C*). This phase transition is accompanied by a steep magnetization jump (21–23) and giant magnetostriction (24) followed by a robust half-magnetization plateau, which can be explained in the framework of microscopic magnetoelastic theories incorporating local phonon modes (25, 26). These findings have triggered further studies on the spin–lattice-coupling physics for various spin models (27–33).

**Fig. 2.**
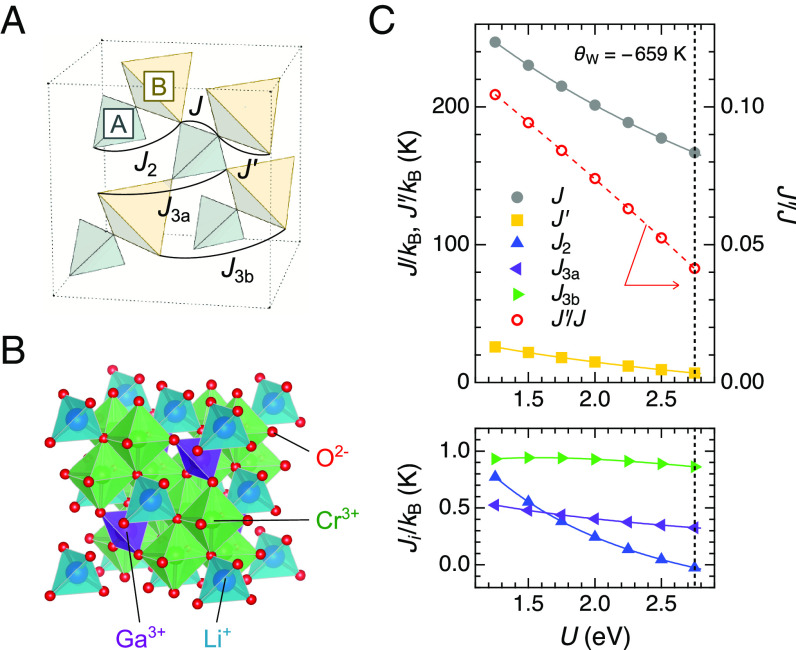
Basic properties of a breathing pyrochlore antiferromagnet LiGaCr_4_O_8_. (*A*) Breathing pyrochlore lattice with two kinds of the nearest-neighbor AFM exchange couplings of *J* (*J′*) in tetrahedra *A* (*B*) with the bond length *r* (*r′*) and further-neighbor exchange couplings up to third nearest neighbor. In LiGaCr_4_O_8_, *r*= 2.907 Å and *r′* = 2.919 Å at 20 K. (*B*) Crystal structure of LiGaCr_4_O_8_. The illustrations are drawn with VESTA software (41). (*C*) Exchange couplings of LiGaCr_4_O_8_ in the paramagnetic state at 20 K determined by DFT energy mapping as a function of on-site interaction strength *U*. The vertical line indicates the *U* value where the exchange couplings match the experimental Weiss temperature *Θ*_W_ = –659 K (36). The resulting exchange parameters are *J*/*k*_B_ = 166.6(2) K, *J′*/*k*_B_ = 6.9(2) K, *J*_2_/*k*_B_ = 0.0(1) K, *J*_3a_/*k*_B_ = 0.3(1) K, and *J*_3b_/*k*_B_ = 0.9(1) K.

Recently, the introduction of a breathing anisotropy, i.e., a spatial modulation of magnetic interactions, has been recognized as a new approach to control the ground state of various spin models (34–36). In this context, the emergence of spin–lattice-coupled superstructures in addition to the conventional 2-up-2-down and 3-up-1-down states (not superstructures) is theoretically proposed in the breathing pyrochlore lattice where the neighboring tetrahedra differ in size in an alternating pattern (Fig. 2*A*) (32). Here, we report the possible realization of this theoretical prediction in a model compound of the breathing pyrochlore antiferromagnet, LiGaCr_4_O_8_ (36–40). By means of state-of-the-art magnetization and magnetostriction measurement techniques available in ultrahigh magnetic fields of up to 600 T, we demonstrate that LiGaCr_4_O_8_ exhibits a two-step magnetostructural transition in an intermediate field range between 150 T and 200 T, followed by a half-magnetization plateau up to ∼420 T. The effective spin Hamiltonian for LiGaCr_4_O_8_ is established based on density-functional-theory (DFT) calculations and classical Monte Carlo (MC) simulations. We show that the combination of the strong spin–lattice coupling and large breathing anisotropy can be responsible for stabilizing the intermediate-field phase, which we assign to a tetrahedron-based superstructure with a three-dimensional periodic array of 3-up-1-down and canted 2-up-2-down spin molecules.

## Results

### Breathing Pyrochlore Antiferromagnet LiGaCr_4_O_8_.

We first introduce the basic properties of the present target LiGaCr_4_O_8_ (36). The crystallographic ordering of nonmagnetic cations Li^+^ and Ga^3+^ identical to the zinc-blende structure (Fig. 2*B*) leads to the breathing pyrochlore Cr network, as shown in Fig. 2*A*. The resultant two inequivalent nearest-neighbor Cr–Cr bonds, whose lengths are *r* = 2.970 Å and *r′* = 2.867 Å at room temperature (36), are characterized by two distinct AFM exchange couplings *J* and *J′*:

[1] $$H_{0}=J\sum_{{\langle i,j\rangle }_{A}}S_{i}\cdot S_{j}+J^{\prime }\sum_{{\langle i,j\rangle }_{B}}S_{i}\cdot S_{j},$$

where

**S***_i_* and **S***_j_* denote the classical Heisenberg spins, and 〉*i*, *j*〈*_A_* and 〉*i*, *j*〈*_B_* stand for nearest-neighbor sites within tetrahedra *A* and *B*, respectively (Fig. 2*A*). Note that *J* is equal to *J′* for the conventional Cr spinel *A*Cr_2_O_4_. For LiGaCr_4_O_8_, *J′* was initially believed to be stronger than *J* (36) judging from the empirical relationship between the strength of the AFM exchange coupling and the lattice constant (equivalently, the Cr–Cr bond length) in *A*Cr_2_O_4_ (42), though the actual situation does not seem so simple. From the structural point of view, the inversion symmetry breaking at the local Cr site would give rise to the anisotropic deformation of Cr 3*d*^3^ orbitals. Indeed, previous DFT energy mapping revealed the dominance of *J* at room temperature: *J*/*k*_B_ = 100 K and *J′*/*k*_B_ = 66.2 K (43), *k*_B_ being the Boltzmann constant. Furthermore, the breathing anisotropy *J′*/*J* can be strongly temperature dependent as exemplified in a related compound LiInCr_4_O_8_ (43).

In order to evaluate *J′*/*J* for LiGaCr_4_O_8_ at low temperatures, we measured powder X-ray diffraction patterns at 20 K and performed the Rietveld analysis (*SI Appendix*, Note 1) and DFT energy mapping (*Materials and Methods*). Since the coexistence of tetragonal and cubic phases are reported below the ordering temperature *T*_N_ = 14 K (38) (*SI Appendix*, Note 2), we can expect reliable information on the exchange parameters from the structural refinement at a temperature slightly higher than *T*_N_. Notably, tetrahedra *A* and *B* are found to be reversed in size (*r* = 2.907 Å and *r′* = 2.919 Å) while the cubic *F*-43*m* structure is preserved at 20 K. Fig. 2*C* shows the DFT energy mapping up to the third nearest-neighbor exchange couplings (Fig. 2*A*). Here, the experimental Weiss temperature *Θ*_W_ = –659 K (36) is used to fix the on-site interaction strength *U*. We find a significant reduction of *J′*/*J* on cooling: *J′*/*J* = 0.66 at room temperature (43) whereas *J′*/*J* = 0.04 at 20 K (*J*/*k*_B_ = 166.6 K and *J′*/*k*_B_ = 6.9 K), indicating that LiGaCr_4_O_8_ is located close to the limit of decoupled AFM tetrahedra at low temperatures. The estimation of *J′*/*J* is nearly independent of *U* (*J′*/*J* = 0.04 ∼ 0.10 for *U* = 1.25 ∼ 2.75 eV), and the second and third nearest-neighbor exchange couplings are negligibly weak.

### Physical Property Measurements in Ultrahigh Magnetic Fields.

**Fig. 3.**
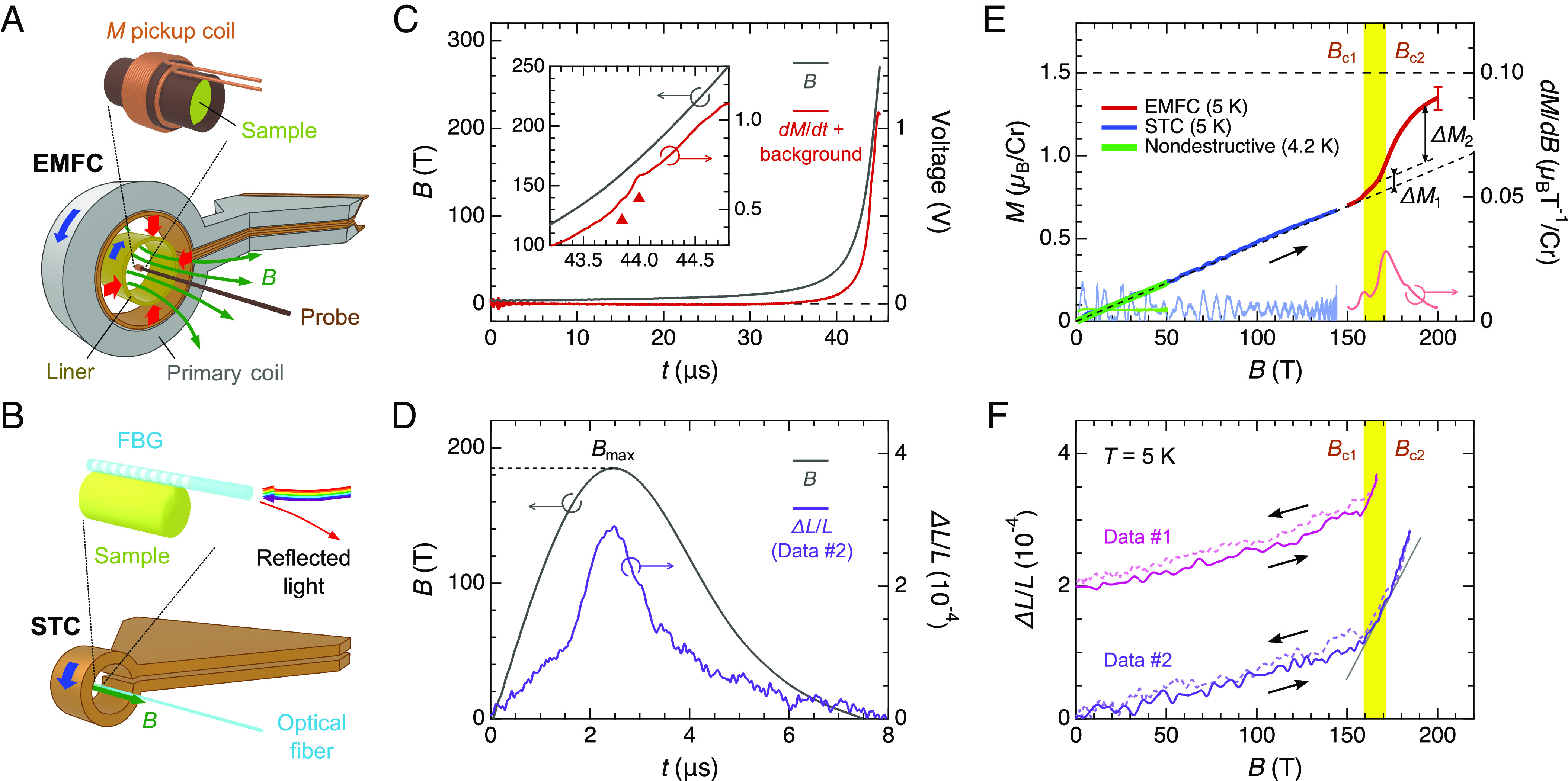
Observation of a two-step magnetostructural transition in LiGaCr_4_O_8_ at around 200 T. (*A*) Schematic of the magnetization measurement using the induction method in the EMFC system. An instantaneous high-current discharge into the outer primary coil induces explosive implosion of the inner brass cylinder (noted as “Liner”) and as a result compresses the magnetic flux (44). (*B*) Schematic of the magnetostriction measurement using the FBG method in the STC system. The probe and sample remain intact during the field pulse due to outward explosion of the STC (45). (*C*) Temporal evolutions of the magnetic field (gray) and the induction voltage detected by the self-compensated pickup coil (red) at 5 K in the EMFC system. The induced voltage is a summation of the intrinsic *dM*/*dt* component and the background originating from *dB*/*dt*. The inset displays an enlarged view around phase transitions denoted by upward triangles in the *dM*/*dt* data. (*D*) Temporal evolutions of the magnetic field (gray) and the sample-length change *∆L*/*L* (purple) obtained in the STC system (corresponding to Data #2). (*E*) *M*–*B* curves (left axis) and their field-derivatives (right axis) at ∼5 K obtained in the nondestructive pulsed magnet (green), STC (blue), and EMFC system (red). The parallel dashed lines are guides for the eye to visualize magnitudes of magnetization jumps *∆M*_1_ and *∆M*_2_ at *B*_c1_ and *B*_c2_, respectively. (*F*) *∆L*/*L*–*B* curves at 5 K obtained in the STC system. Data #1 and #2 were taken in the same sample setting with different generated maximum field *B*_max_ (Data #1 is vertically shifted for clarity). The gray solid line is a guide for the eye to visualize the slope change in *∆L*/*L* at *B*_c1_ and *B*_c2_.

As suggested from the large negative Weiss temperature *Θ*_W_ = –659 K (36), ultrahigh magnetic fields of several hundreds of tesla are required to exceed the strong AFM exchange interactions in LiGaCr_4_O_8_. Accordingly, we employed two kinds of destructive-type pulsed megagauss generators: an electromagnetic flux compression (EMFC) system (44) (Fig. 3*A*) and a single-turn coil (STC) system (45) (Fig. 3*B*). The former allows physical property measurements up to ∼600 T only in the field-increasing process, whereas the latter offers the data up to ∼200 T both in the field-increasing and -decreasing processes. Typical magnetic-field waveforms generated by the EMFC and STC systems are shown by gray lines in Fig. 3 *C* and *D*, respectively.

Physical property measurements on magnetic insulators in the EMFC system were so far limited to the Faraday rotation and optical absorption measurements (23). These magneto-optical detection schemes, however, could not be applied to the present polycrystalline LiGaCr_4_O_8_ samples. In this work, we have extended two existing techniques implemented in the STC system to the EMFC system to observe successive phase transitions of LiGaCr_4_O_8_ up to ∼600 T: i) a magnetization measurement using the induction method (31, 46, 47) and ii) a magnetostriction measurement using the optical fiber-Bragg-grating (FBG) method (48, 49) (*SI Appendix*, Notes 3 and 6).

### Two-Step Magnetostructural Transition at around 200 T.

Fig. 3*E* summarizes the magnetization data of LiGaCr_4_O_8_ measured at ∼5 K. In the STC system, a linear increase in the magnetization *M* with respect to the external magnetic field *B* is observed up to a generated maximum field *B*_max_ of 145 T, where *M* reaches 0.70 *μ*_B_/Cr, suggesting that spins are smoothly canting from the 2-up-2-down AFM ground state (38). Here, the absolute value of *M* is calibrated using the magnetization data obtained in a SQUID magnetometer MPMS up to 7 T (*SI Appendix*, Note 2) and in a nondestructive pulsed magnet up to 51 T (Fig. 3*E*). Upon the application of a higher field using the EMFC system, *dM*/*dt* anomalies with a small hump and a subsequent large hump are observed at *B*_c1_ = 159 T and *B*_c2_ = 171 T, respectively (*Inset* of Fig. 3*C*), indicating a two-step metamagnetic transition. We ensure the reproducibility in three independent experiments with different setups (*SI Appendix*, Note 4). By subtracting the background component from the observed *dM*/*dt* profile (*SI Appendix*, Note 5), *M* as a function of *B* and its field derivative *dM*/*dB* between 150 T and 200 T are obtained as shown by red and pink lines, respectively, in Fig. 3*E*. The second magnetization jump, *ΔM*_2_, is approximately six times larger than the first one, *ΔM*_1_, if the *B*-linear component is subtracted (Fig. 3*E*). The absolute value of *M* at 200 T is 1.35 ± 0.07 *μ*_B_/Cr, which is close to half the expected saturation magnetization of ∼3 *μ*_B_/Cr given that the *g*-value is estimated to be *g* = 1.98 ∼ 2.08 (36, 37). The present observation is most likely ascribed to the appearance of a half-magnetization plateau as observed in *A*Cr_2_O_4_ (21–23), given that the magnetization jump is blunted by thermal fluctuations or crystallographic disorders.

The existence of an intermediate-field phase between *B*_c1_ and *B*_c2_ is supported by the magnetostriction measurements. Fig. 3*F* shows the magnetostriction data of LiGaCr_4_O_8_ measured at 5 K using the STC system with *B*_max_ = 167 T (Data #1) and 185 T (Data #2). The temporal evolution of the relative sample-length change *∆L*/*L* for Data #2 is shown in Fig. 3*D*. Data #1 and #2 were taken in the same setup and are in excellent agreement with each other. As seen in Data #1, a rapid lattice expansion starts at around 160 T, which corresponds to the phase transition at *B*_c1_. Furthermore, as seen in Data #2, the increase in *∆L*/*L* is accelerated above *B*_c2_. These observations indicate that both transitions at *B*_c1_ and *B*_c2_ are likely first order accompanied by a structural phase transition.

### Field Width of Half-Magnetization Plateau.

Theoretically, the magnetic-field width of a half-magnetization plateau correlates with the strength of the spin–lattice coupling in the (breathing) pyrochlore-lattice Heisenberg antiferromagnet (25, 32). Therefore, it is important to determine the termination field of the half-magnetization plateau *B*_c3_ for further discussions, although our magnetization measurement could not detect additional phase transitions above 200 T due to poor sensitivity of our probe in the high-field region (*SI Appendix*, Note 4).

**Fig. 4.**
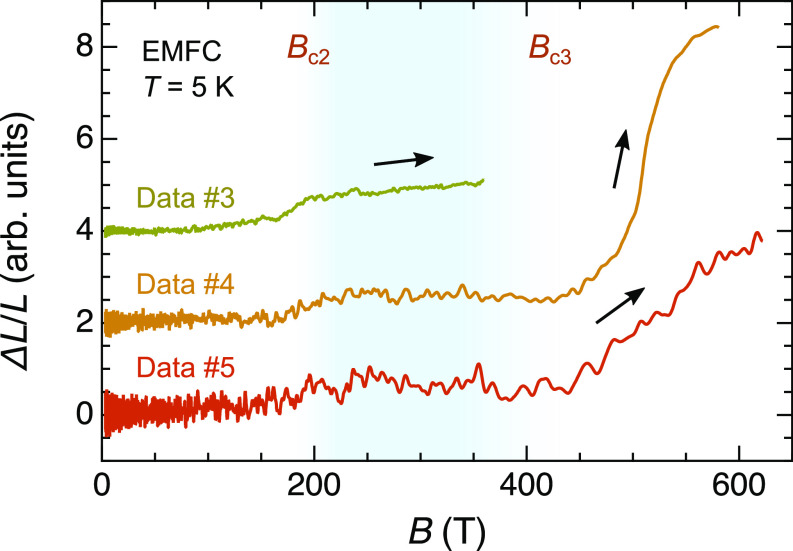
Magnetic-field width of a half-magnetization plateau in LiGaCr_4_O_8_ determined by the magnetostriction measurements. *∆L*/*L*–*B* curves at 5 K obtained in the EMFC system. Data #3, #4, and #5 were taken in independent sample settings with the generated maximum field of *B*_max_ = 360, 580, and 620 T, respectively (Data #3 and #4 are vertically shifted for clarity). The half-magnetization plateau associated with the 3-up-1-down magnetic state is expected to appear in a field region shaded by cyan.

As an alternative way to determine *B*_c3_, we measured the magnetostriction of LiGaCr_4_O_8_ at 5 K in the EMFC system. Considering the relation *∆L*/*L* ∝ *M*^2^ derived from the magnetoelastic theory (24), there should be no lattice-constant change in the half-magnetization plateau region, followed by a significant volume expansion on the high-field side. Fig. 4 shows three *∆L*/*L*–*B* curves (Data #3 ∼ #5) obtained in independent sample settings. The raw data are shown in *SI Appendix*, Note 7, and the analytical procedure is described in *SI Appendix*, Note 8. For Data #3, a plateau-like behavior is observed from 200 T up to *B*_max_ = 360 T. For Data #4 and #5 with *B*_max_ ≈ 600 T, on the other hand, an upturn behavior is clearly observed at around 420 T, signaling the occurrence of a phase transition from the 3-up-1-down to a higher-field spin-canted phase. We hence determine *B*_c3_ ≈ 420 T. Furthermore, another *∆L*/*L* kink is observed at around 550 T, potentially reflecting a phase transition to a paramagnetic phase.

### Classical Monte Carlo Simulations.

To get more insights into the field-induced phase transitions of LiGaCr_4_O_8_, we employ a microscopic magnetoelastic model on the breathing pyrochlore lattice (32) incorporating the Einstein site phonons assuming the independent displacement of each site (26). The effective spin Hamiltonian *H*_eff_ is

[2] $$H_{eff}=H_{0}+H_{SLC}-h\sum_{i}S_{i},$$

where $H_{0}$ contains minimal exchange terms expressed as Eq. **1**, *h* is the external magnetic field applied along the *z*-axis, and

[3] $$\begin{array}{ll} H_{\text{SLC}} & =-Jb\sum_{{\langle i,j\rangle }_{A}}{\left(S_{i}\cdot S_{j}\right)}^{2}-J^{\prime }b^{\prime }\sum_{{\langle i,j\rangle }_{\text{B}}}{\left(S_{i}\cdot S_{j}\right)}^{2}\\ & -\sum_{i}\left\{\frac{Jb}{4}\sum_{j\neq k\in N_{A}\left(i\right)}+\frac{J^{\prime }b^{\prime }}{4}\sum_{j\neq k\in N_{B}\left(i\right)}\right\}\left(S_{i}\cdot S_{j}\right)\left(S_{i}\cdot S_{k}\right)\\ & -\sqrt{JJ^{\prime }bb^{\prime }}\sum_{i}\sum_{j\in N_{A}\left(i\right)}\underset{k\in N_{B}\left(i\right)}{\sum }e_{ij}\cdot e_{ik}\left(S_{i}\cdot S_{j}\right)\left(S_{i}\cdot S_{k}\right), \end{array}$$

where *b* (*b′*) is a dimensionless parameter representing the strength of the spin–lattice coupling between the nearest-neighbor sites within tetrahedra *A* (*B*), *N_A_*(*i*) (*N_B_*(*i*)) denotes a set of the nearest-neighbor sites of site *i* within tetrahedra *A* (*B*), and **e***_ij_* is the unit vector oriented from site *i* to *j*. Possible effects from the single-ion anisotropy can be neglected here because of the quenched orbital angular momentum of the Cr^3+^ ion, as indicated by the *g*-value [*g* = 1.98 ∼ 2.08 (36, 37)]. In fact, the magnetization curves of CdCr_2_O_4_ [*g* = 2.06 (17)], for example, for *H* ∥ [100], [110], and [111] are almost identical (18). As for phonon contributions, the displacement energies of atoms are quadratic in the Hamiltonian so that they can be exactly traced out through the standard Gaussian integration (for the derivation process of Eq. **3**, see *SI Appendix*, Note 9). Consequently, the phonon-mediated spin interactions $H_{SLC}$ consist of biquadratic terms favoring collinear spin configurations (first and second terms) and three-body quartic terms responsible for lifting the macroscopic degeneracy (third and last terms). Note that Eq. **2** with *J′*/*J* = 1 can reproduce a half-magnetization plateau with a 16-sublattice 3-up-1-down magnetic structure observed in HgCr_2_O_4_ (19) and CdCr_2_O_4_ (20), where up-up-up-down chains run along all the six equivalent [110] directions (26). The magnetic phase diagrams for the moderately breathing case of *J′*/*J* = 0.6 and 0.2 are reported in ref. 32.

**Fig. 5.**
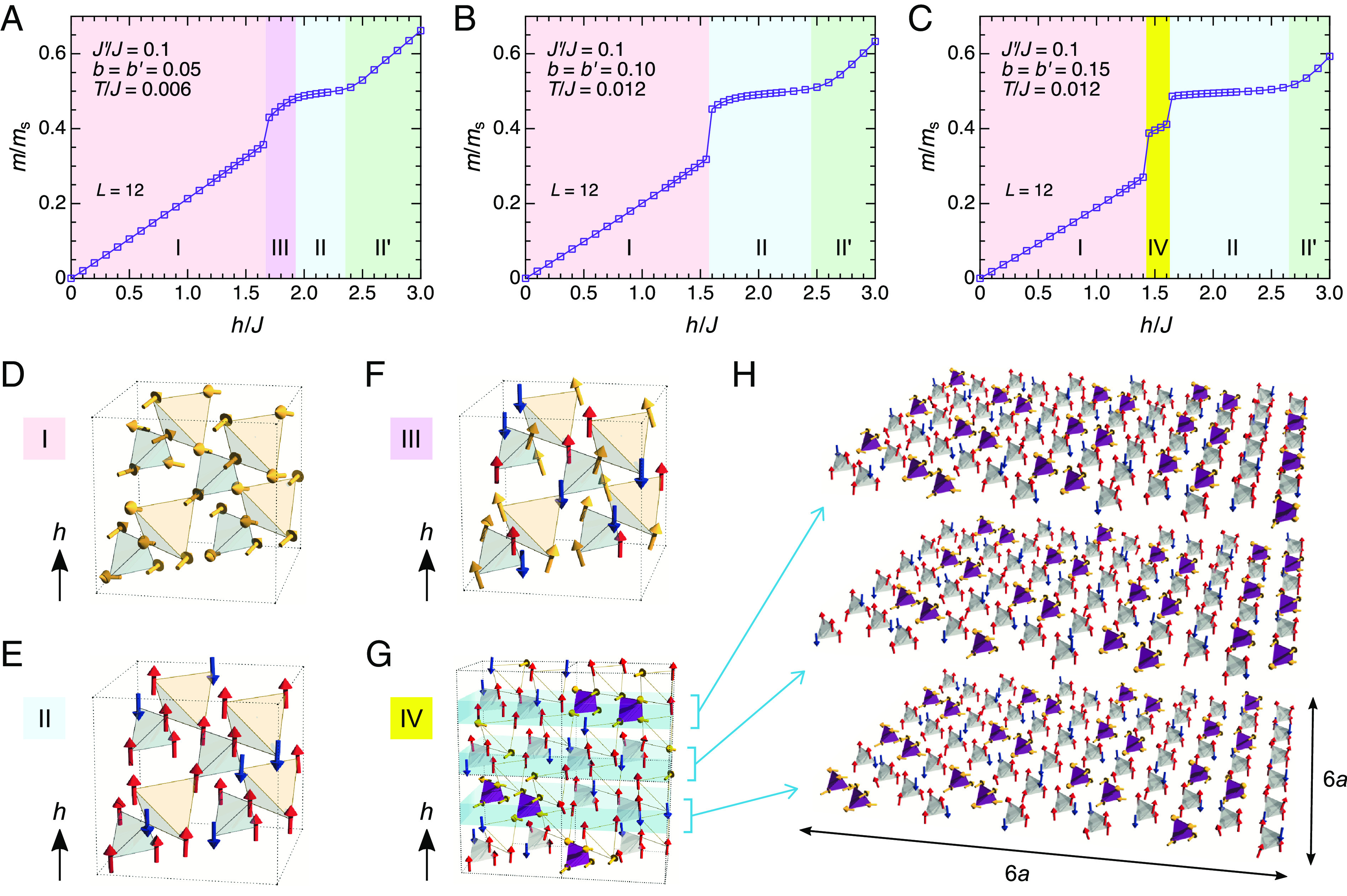
Successive field-induced phase transitions based on the magnetoelastic theory on the breathing pyrochlore Heisenberg antiferromagnet. (*A*–*C*) Magnetization curves in the strongly breathing case of *J′*/*J* = 0.1 for (*A*) *b* = *b′* = 0.05, *T*/*J* = 0.006, (*B*) *b* = *b′* = 0.10, *T*/*J* = 0.012, and (*C*) *b* = *b′* = 0.15, *T*/*J* = 0.012 calculated by means of the classical MC simulations. (*D*–*H*) Real-space magnetic structures in (*D*) phase I, (*E*) II, (*F*) III, and (*G* and *H*) IV, where red (blue) and yellow arrows represent spins pointing upward (downward) and canting with respect to the magnetic field *h*, respectively. In phases I ∼ III, the canted 2-up-2-down, 3-up-1-down, and "1-up-1-down + V"-type spin configurations are stabilized, respectively, with no translational symmetry breaking of the underlying breathing pyrochlore lattice. In phase IV, a tetrahedron-based long-range order with a sixfold magnetic unit cell size in all the three principle axes emerges. In *H*, the spin configurations within three adjacent tetrahedral layers are displayed (corresponding to one-fourth of the magnetic unit cell). This magnetic state is composed of the canted 2-up-2-down (purple) and 3-up-1-down tetrahedra (gray) in the ratio of 1:2.

As mentioned above, LiGaCr_4_O_8_ is characterized by a large breathing anisotropy of *J′*/*J* = 0.04 just above *T*_N_ according to the DFT calculations, although the situation would be more complex below *T*_N_ (38). Here, we set *J′*/*J* = 0.1 as a typical value for the strongly breathing case in Eq. **2** and performed classical MC simulations to calculate the magnetization curves for various sets of the spin–lattice coupling parameters *b* and *b′*. Fig. 5 *A*–*C* shows the calculation results for a system size of *N* = 16*L*^3^ spins with *L* = 12 in three typical cases: a) *b* = *b′* = 0.05, b) *b* = *b′* = 0.10, and c) *b* = *b′* =0.15. Regardless of *b* and *b′*, phase I with an 8-sublattice canted 2-up-2-down state (Fig. 5*D*) and phase II with a 16-sublattice 3-up-1-down state (Fig. 5*E*) appear followed by a higher-field phase II’ (the magnetic structure of phase II’ is likely to be more complicated than a simple canted 3-up-1-down state, and its identification is beyond the scope of this work). An important difference manifests just below phase II. In the weak spin–lattice coupling case of *b* = *b′* = 0.05, the system undergoes a first-order transition from phase I to III with a 16-sublattice "1-up-1-down+V"- type spin correlation (Fig. 5*F*), which is continuously connected to phase II. With increasing the spin–lattice coupling, an intermediate-field phase once disappears for *b* = *b′* = 0.10, and then another phase, phase IV, appears for *b* = *b′* = 0.15. Remarkably, the magnetic structure of phase IV is a mixture of the canted 2-up-2-down and 3-up-1-down units of tetrahedra *A* in the ratio of 1:2, forming a long-range order with a 6 × 6 × 6 magnetic unit cell (Fig. 5 *G* and *H*). The detail of the ordering pattern is described in ref. 32. We confirm that, for system sizes in which *L* is not a multiple of six, this state cannot be obtained, and instead a metastable state with higher energy appears (*SI Appendix*, Note 10). Note that phase IV appears also for *J′*/*J* = 0.2 with larger values of *b* and *b′* (*b* = *b′* ≳ 0.20) but does not appear for *J′*/*J* = 0.6 with any values of *b* and *b′* (32).

## Discussion

From the above results, we find that the theoretical *M*–*B* curve for the strong spin–lattice coupling case of *b* = *b′* = 0.15 (Fig. 5*C*) is qualitatively compatible with the experimental *M*–*B* curve of LiGaCr_4_O_8_ (Fig. 3*E*) in that the two-step magnetization jump as well as the relatively wide half-magnetization plateau are reproduced. We hence believe that the experimentally observed intermediate-field phase prior to the half magnetization plateau would be a magnetic superstructure phase induced by the strong spin–lattice coupling and large breathing anisotropy. However, the magnetization in phase IV amounts to *m*/*m*_s_ ≈ 0.4 (Fig. 5*C*), which is larger than the experimental value at *B*_c2_, *m*/*m*_s_ ≈ 0.3 (Fig. 3*E*). Moreover, the present site-phonon model tends to underestimate the field width of the half-magnetization plateau (26, 32), as demonstrated in *A*Cr_2_O_4_ with *J′*/*J* = 1 (21, 22) and also in the present case (*SI Appendix*, Note 11). These quantitative discrepancies may arise from the missing incorporation of the macroscopic lattice deformation in the site-phonon model. Another microscopic magnetoelastic model assuming independent bond-length changes, i.e., the bond-phonon model, can resolve this issue (*SI Appendix*, Note 11), although it can describe neither magnetic long-range orders nor additional complicated magnetic transitions found in the site-phonon model (25, 32). The development of an extended magnetoelastic Hamiltonian is necessary to fill the gap between experiment and theory.

Finally, it is worth mentioning the relationship of the present findings to the magnetism of isolated tetrahedral clusters, namely *J′* = 0. If we take into account quantum spins and neglect the spin–lattice coupling, the application of a magnetic field induces the quantization of a total spin number per cluster, which can be regarded as a spin crossover rather than a phase transition. As a consequence, the *M*–*B* curve exhibits fractional magnetization plateaus at *m*/*m*_s_ = *p*/2*n* for spin-*n*/2 systems (*p*, *n*: integer values satisfying 0 < *p* < 2*n*). Such a magnetization behavior was indeed observed in a spin-1/2 breathing pyrochlore antiferromagnet Ba_3_Yb_2_Zn_5_O_11_ (50) and a spin-3/2 tetrahedral-cluster compound Co_4_B_6_O_13_ (51). In LiGaCr_4_O_8_, however, no plateau-like structure is observed at around *m*/*m*_s_ = 1/6, i.e., *M* ≈ 0.5 *μ*_B_/Cr (Fig. 3*E*), suggesting that a spin crossover is masked by the intertetrahedral exchange coupling *J′*. Also, it is obvious that if we set *J′* = 0 and *b′* = 0 in the classical Heisenberg model with the spin–lattice coupling Eq. **2**, no magnetic superstructure phase appears due to the absence of spin interactions within tetrahedra *B*. Three-body spin interactions across tetrahedra *A* and *B* (Fig. 2*A*) originating from the site-dependent local phonons, corresponding to the last term in Eq. **3**, play a crucial role in bringing about rich field-induced phases in the breathing pyrochlore system.

In summary, we observe a two-step magnetostructural transition between 150 T and 200 T prior to a robust half-magnetization plateau in the breathing pyrochlore antiferromagnet LiGaCr_4_O_8_. Considering the magnetoelastic theory incorporating local phonon modes, the intermediate-field phase can be assigned to a spin–lattice-coupled superstructure with a three-dimensional periodic array of 3-up-1-down and canted 2-up-2-down spin molecules, which we attribute to the strong spin–lattice coupling and large breathing anisotropy. The present work, combining the exotic experimental observations with the microscopic magnetoelastic theory in the complicated three-dimensional frustrated magnet, paves the way for further verifications of intriguing physical phenomena originating from the spin–lattice coupling and/or breathing anisotropy, both of which can be relevant in magnetic materials regardless of the geometry of the underlying crystalline lattice. For instance, the formation of a 3 × 3 spin–lattice-coupled superstructure phase associated with a 1/9-magnetization plateau as well as a $\sqrt{3}$ × $\sqrt{3}$ one associated with a 1/3-magnetization plateau is theoretically predicted in a kagome-lattice Heisenberg antiferromagnet (33); its experimental verification as well as theoretical studies on the effect of introducing the breathing anisotropy remain open questions. In addition, we demonstrate that the electromagnetic induction and FBG methods are powerful tools to detect magnetic and structural phase transitions, respectively, in ultrahigh magnetic fields well above 100 T. These techniques would be applicable to a broad range of materials such as frustrated magnets, spin-crossover systems, heavy-fermion compounds, and superconductors, leading to further flourishing of high magnetic field science.

## Materials and Methods

### Sample Preparation.

Polycrystalline samples of LiGaCr_4_O_8_ were obtained from the same batch used in ref. 36, prepared by the conventional solid-state reaction method. The powder samples were formed into rod shape with ∼0.8 mm diameter and ∼1.5 mm length using epoxy (Stycast 1266) for the magnetostriction measurement.

### Powder X-ray Diffraction Measurement and Structural Analysis.

We performed the powder X-ray diffraction measurement on LiGaCr_4_O_8_ at 20 K using a commercial X-ray diffractometer (SmartLab, Rigaku). The incident X-ray beam was monochromated by a Johansson-type monochromator with a Ge(111) crystal to select only Cu-*Kα*1 radiation. Only a tiny amount of impurity phases were found, ensuring that the sample is of high quality. The Rietveld analysis was performed using the JANA2006 program (52), confirming the cubic *F*-43*m* structure. Detailed results are shown in *SI Appendix*, Note 1.

### DFT Calculations.

Exchange parameters of LiGaCr_4_O_8_ were estimated by the DFT-based energy mapping (53, 54). We performed all electron DFT calculations using the full potential local orbital code (55). Note that this technique has proven to be very reliable for the breathing pyrochlore chromium spinels (31, 43). We used the generalized gradient approximation (GGA) exchange and correlation functional (56). For the electronic structure calculations, we used the *T* = 20 K crystal structure with the *F*-43*m* space group determined in this work. We accounted for strong correlations on the Cr 3*d* orbitals by applying a GGA+*U* exchange correlation functional (57) for several different values of *U* and *J*_H_ = 0.72 eV fixed following ref. 58.

### Magnetization and Magnetostriction Measurements.

The magnetization up to 7 T was measured using a SQUID magnetometer (MPMS; Quantum Design). The magnetization up to 51 T, 145 T, and 200 T was measured by the induction method in a nondestructive pulsed magnet, a horizontal STC system (45), and an EMFC system (44), respectively. The details of experimental setup, data, and analysis method for the magnetization measurements using the EMFC system are presented in *SI Appendix*, Notes 3–5, which include additional references (59–63). The longitudinal magnetostriction up to 185 T and ∼600 T was measured by the optical FBG method in the horizontal STC and EMFC systems, respectively. Here, a relative sample-length change *∆L*/*L* was detected by the optical filter method with the resolution of 10^–5^ to 10^–4^ (48, 49). The details of experimental setup, data, and analysis method for the magnetostriction measurements using the EMFC system are presented in *SI Appendix*, Notes 6–8. All of the experiments were performed at the Institute for Solid State Physics, University of Tokyo, Kashiwa, Japan.

### Classical Monte Carlo Simulations.

To identify various magnetic phases appearing in the spin Hamiltonian Eq. **2**, we performed classical Monte Carlo (MC) simulations in which a spin vector at each lattice site is updated in conventional random and successive over-relaxation-like processes. In the former (latter) process, we tried to rotate a spin in a randomly proposed direction (by the angle π around the local mean field) by using the Metropolis algorithm. In our simulation, 2 × 10^6^ MC site sweeps were carried out at each temperature and magnetic field under the periodic boundary condition, and the first half was discarded for thermalization. Observations were done in every 10 MC sweeps and the statistical average was taken over four independent runs. We often encountered various metastable states due to very complicated interactions in the spin–lattice coupling term Eq. **3**, and low-temperature spin states obtained in the field-cooling, field-increasing, and field-decreasing processes were sometimes different. In such a situation, we compare the thermal average values of the energy of these states and regard the lowest-energy state as the equilibrium state. Since our cubic unit cell contains 16 sites, the total number of spins *N* is related to the linear system size *L* via *N* = 16*L*^3^. We have checked that the results for *L* = 6 and 12 are essentially the same and consistent with those for smaller *L*’s, e.g., *L* = 1, where the thermalization is much easier (the spin configurations for phases I, II, and III shown in Fig. 5 can be described even in the small-size system of *L* = 1), so that only the result for *L* = 12 is shown in Fig. 5 *A*–*C*.

## Supplementary Material

Appendix 01 (PDF)

## Data Availability

All study data are included in the article and/or *SI Appendix*.
