# Supplementary material for: Signatures of a magnetic superstructure phase induced by ultrahigh magnetic fields in a breathing pyrochlore antiferromagnet
Source: Proc Natl Acad Sci U S A. 2023 Aug 7;120(33):e2302756120. doi: 10.1073/pnas.2302756120 (PMC10438373; doi:10.1073/pnas.2302756120)
Supplement: Supplementary file 1 — Appendix 01 (PDF) [file pnas.2302756120.sapp.pdf]

Supplementary Information for
"Signatures of a magnetic superstructure phase induced by ultrahigh magnetic fields in a breathing pyrochlore antiferromagnet"

M. Gen,[1,2,*] A. Ikeda,[3,4] K. Aoyama,[5] H. O. Jeschke,[6] Y. Ishii,[1] H. Ishikawa,[1] T. Yajima,[1] Y. Okamoto,[1] X.-G. Zhou,[1] D. Nakamura,[1,2] S. Takeyama,[1] K. Kindo,[1] Y. H. Matsuda,[1] and Y. Kohama[1,†]

[1]*Institute for Solid State Physics, University of Tokyo, Kashiwa, Chiba 277-8581, Japan*
[2]*RIKEN Center for Emergent Matter Science (CEMS), Wako, Saitama 351-0198, Japan*
[3]*Institute for Solid State Physics, University of Tokyo, Kashiwa 277-8581, Japan*
[4]*Department of Engineering Science, University of Electro-Communications, Chofu, Tokyo 182-8585, Japan*
[5]*Department of Earth and Space Science, Graduate School of Science, Osaka University, Osaka 560-0043, Japan*
[6]*Research Institute for Interdisciplinary Science, Okayama University, Okayama 700-8530, Japan*

This Supplementary Information includes contents as listed below:

Note 1. Structural analysis of $LiGaCr_4O_8$ at 20 K
Note 2. Magnetizatinon data up to 7 T obtained in MPMS
Note 3. Details of magnetization measurement technique in the EMFC system
Note 4. All the magnetization data obtained in the EMFC system
Note 5. Analysis of the magnetization data obtained in the EMFC system
Note 6. Details of magnetostriction measurement technique in the EMFC system
Note 7. All the magnetostriction data obtained in the EMFC system
Note 8. Analysis of the magnetostriction data obtained in the EMFC system
Note 9. Site-phonon model on the breathing pyrochlore Heisenberg antiferromagnet
Note 10. System size dependence of the calculated results on Monte Carlo simulations
Note 11. Magnetic-field width of the half-magnetization plateau

Note 1. Structural analysis of $LiGaCr_4O_8$ at 20 K

The crystallographic parameters of $LiGaCr_4O_8$ polycrystalline samples used in this study at 293 K was reported in Ref. [1]. Here, we investigated the structural change in the paramagnetic region by measuring the powder X-ray diffraction (XRD) patterns at 20 K using a commercial X-ray diffractometer (SmartLab, Rigaku). Figure S1 shows the observed powder XRD pattern and the result of the Rietveld analysis based on a structural model with the cubic space group $F\bar{4}3m$. The crystallographic parameters obtained by assuming the occupancy of each site to be 1 are summarized in Table S1.

TABLE S1. Crystallographic parameters of $LiGaCr_4O_8$ polycrystalline samples at 20 K. The lattice constant is a = 8.24031(2) Å.

R_p = 5.13%, R_{wp} = 6.13%, S = 1.260						
		x	y	z	Occupancy	$100U_{iso}$ (Å^2)
Li	4a	0	0	0	1	1
Ga	4d	3/4	3/4	3/4	1	0.62(4)
Cr	16e	0.37526(15)	x	x	1	0.54(3)
O1	16e	0.1376(2)	x	x	1	0.78(5)
O2	16e	0.6275(4)	x	x	1	0.78(5)

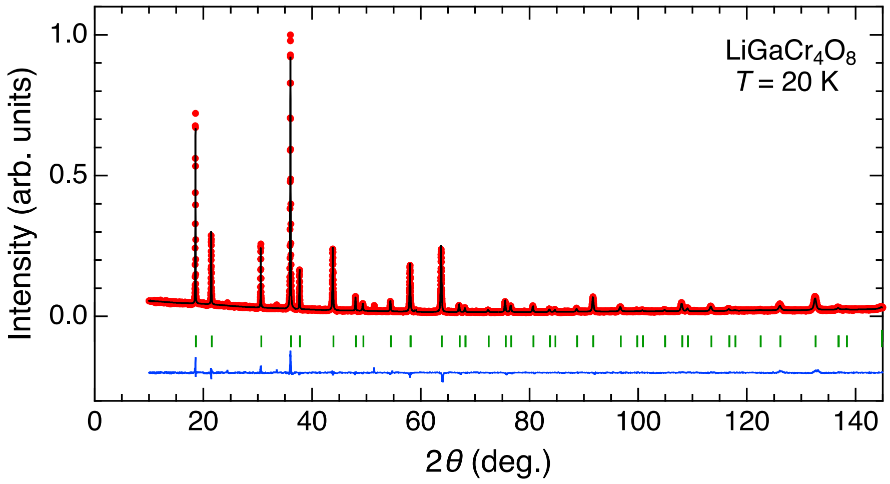

FIG. S1. Refinement result of the powder XRD patterns of $LiGaCr_4O_8$ at 20 K. The red filled circles and the black solid line show the experimental data points and the calculated pattern, respectively. The vertical bars indicate the positions of the structure Bragg peaks. The blue line shows the difference in the experimental and calculated intensities.

Note 2. Magnetization data up to 7 T obtained in MPMS

Figure S2 summarizes the magnetization data of $LiGaCr_4O_8$ polycrystalline samples obtained using a SQUID magnetometer (MPMS; Quantum Design). Figure S2(a) shows the temperature dependence of the magnetic susceptibility χ measured at 1 T and 7 T. χ rapidly drops at $T_N \approx 14$ K at 1 T, indicating an antiferromagnetic (AFM) phase transition. Figure S2(b) shows the magnetization curve measured up to 7 T. A slightly upturn behavior is seen at around 2 T, suggesting a weak spin-flop transition or a crossover. Note that the coexistence of tetragonal and cubic phases are reported in the zero-field AFM state below T_N [2]. The applied magnetic field may favor one of them, resulting in a change in the slope of the M–B curve.

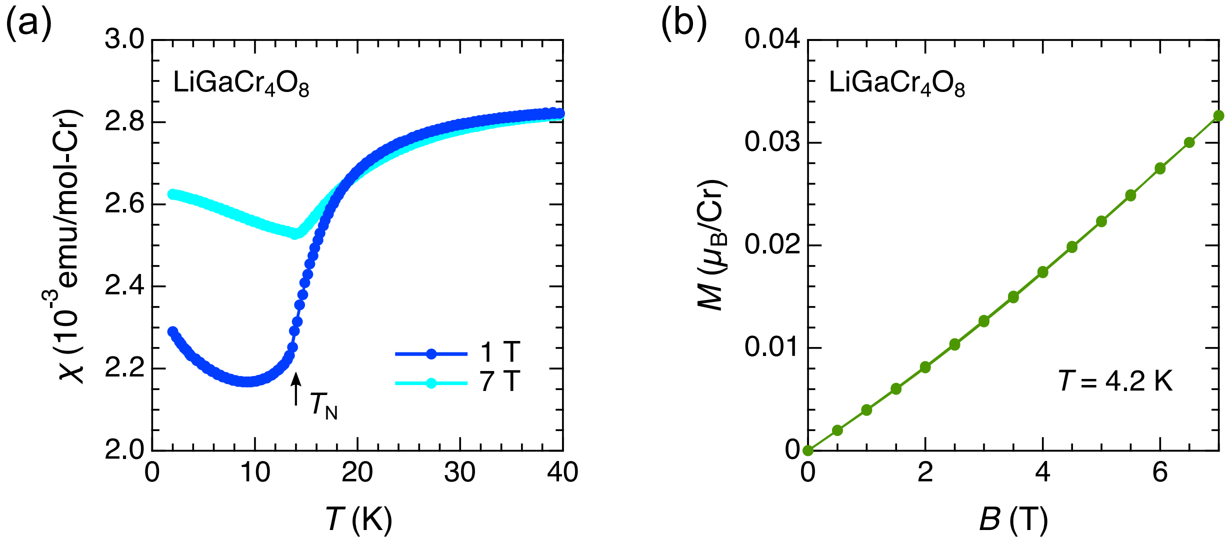

FIG. S2. (a) Temperature dependence of the field-cooled magnetic susceptibility χ of $LiGaCr_4O_8$ measured in a magnetic field of 1 T (blue) and 7 T (cyan). (b) Magnetization curves of $LiGaCr_4O_8$ measured at 4.2 K up to 7 T.

Note 3. Details of magnetization measurement technique in the EMFC system

In many frustrated magnets, the energy scale of the overall AFM exchange interactions reaches several hundreds of kelvin. Therefore, magnetization measurements in ultrahigh magnetic fields above 100 T are indispensable to understand their underlying physics. Over the past 30 years, the electromagnetic induction method using the single-turn coil (STC) system has been improved step by step [3–9], and now precise magnetization measurements up to 150 T are available [10]. However, the induction method in much higher fields using the electromagnetic flux compression (EMFC) system remains very challenging because of (i) the difficulty in the magnetic-field generation itself, (ii) the complete destruction inside the magnet coil by a single shot, and (iii) the poor reproducibility of the magnetic-field waveform. Thus, it is impractical to experimentally subtract the uncompensated dB/dt component from the dM/dt waveform by the conventional way adopted in the non-destructive pulsed magnet and the STC system, i.e., performing the measurement twice with sample-in and sample-out conditions under the same magnetic-field waveform.

For detecting dM/dt signal in the EMFC system, we adopted two types of handmade self-compensated M pickup coils, coaxial-type [Fig. S3(a)] and straight-type [Fig. S3(b)]. These shapes have an advantage compared to the parallel-type [Fig. S3(c)] in that the special inhomogeneity of the generated magnetic field in a radial direction can be suppressed. Indeed, it has been experimentally demonstrated that at above 100 T the field generation space is already smaller than 2 cm in a diameter, and its special inhomogeneity significantly increases in a radial direction [11, 12]. The M pickup coils were made of a polyimide-amide enameled copper wire (AIW wire, TOTOKU Electric Co. Ltd.) with an outer diameter of 0.06 mm and a withstand voltage of ~1 kV, which were wound around a long Kapton tube. The typical designs are as follows: 21 turns (or 29 turns) for an inner coil with a diameter of 1.3 mm and 13 turns (or 18 turns) for an outer coil with a diameter of 1.7 mm for the coaxial-type, and 10 turns for both left and right coils with a diameter of 1.7 mm for the straight-type (see also the photos in Fig. S4). It was confirmed that these M pickup coils achieve a high compensation ratio of more than 99.9 % and can survive without dielectric breakdown until they are mechanically destroyed by the implosion of a liner at around the maximum field of 400–600 T. The magnetic field was simultaneously measured by another B pickup coil wound outside the M pickup coil.

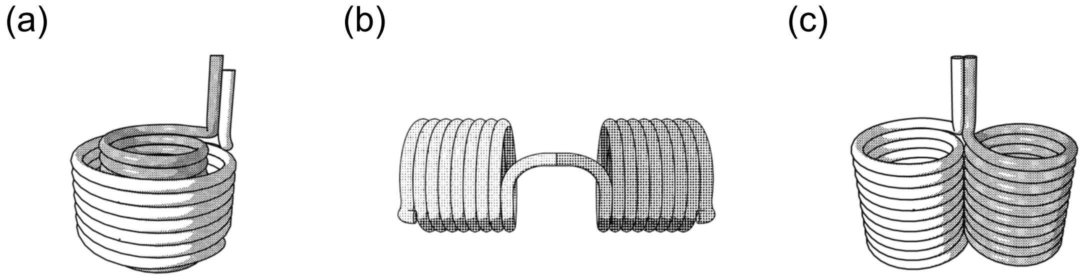

FIG. S3. Schematics of self-compensated M pickup coils with three different geometries: (a) coaxial-type, (b) straight-type, and (c) parallel-type.

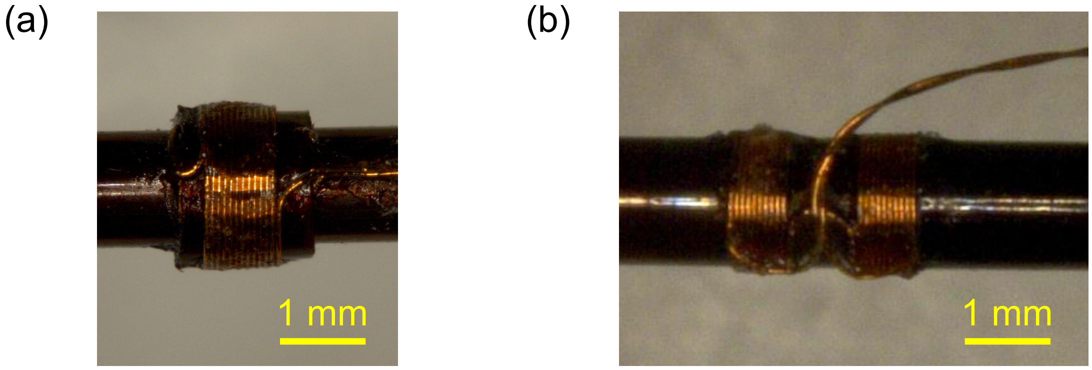

FIG. S4. Photographs of self-compensated M pickup coils: (a) coaxial-type, (b) straight-type.

In order to cool the sample down to ∼5 K, the M and B pickup coils were installed in a double-layerd liquid-^{4}He flow cryostat made of glass-epoxy (G10), as illustrated in Fig. S5. The $LiGaCr_4O_8$ powder samples were packed inside the Kapton tube wound with the M pickup coil in a length of 3 mm and was fixed between sticks made of G10 from both sides. To monitor the sample temperature, a RuO_2 tip thermometer was glued to the Kapton tube in the vicinity of the sample.

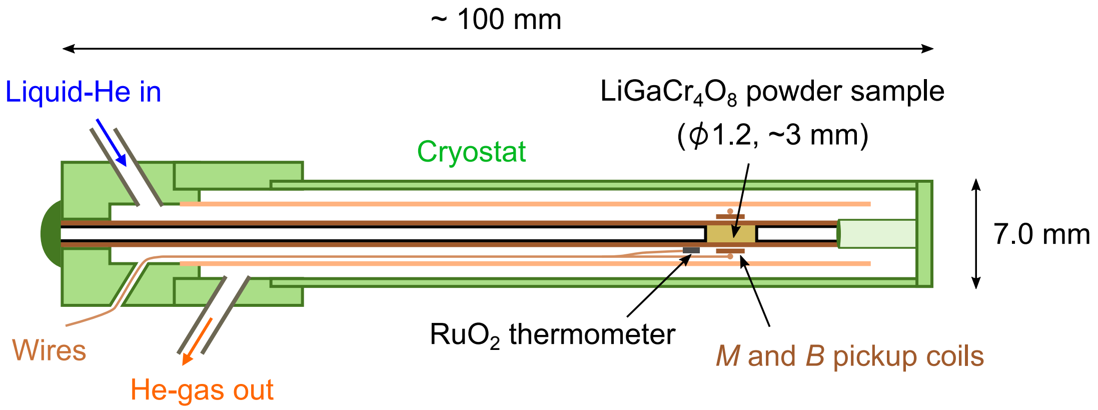

FIG. S5. Cross-sectional view of the liquid-^{4}He flow cryostat equipped with the M and B pickup coils, RuO_2 thermometer, and sample.

Note 4. All the magnetization data obtained in the EMFC system

We performed the magnetization measurement on $LiGaCr_4O_8$ using the EMFC system in total three times. Here, the coaxial-type M pickup coil was employed for the 1st and 2nd experiments, while the straight-type for the 3rd. The temporal evolutions of the magnetic field (gray) and the induction voltage detected by the M pickup coil (red) for each experiment are shown in Figs. S6(a)–S6(c), where the measurement temperature was ~8 K, ~5 K, and ~7 K, respectively. In the main text, we show the data of the 2nd experiment as representative (Figs. 3C and 3E). As denoted by triangles in the insets, the observation of double-hump dM/dt anomalies around 160–170 T are reproduced in all the experiments, indicating that the two-step transition is intrinsic. We note that the background component is much larger and its turnaround behavior occurs at a lower magnetic field in the dM/dt waveform for the 3rd experiment, making the intrinsic dM/dt anomalies blurred. Thus, we conclude that the coaxial-type M pickup coil is superior to the straight-type for detecting the intrinsic M signal sensitively up to as high magnetic fields as possible.

In addition, we also performed the magnetization measurement on $LiInCr_4O_8$ as a reference sample, which is known to exhibit a first-order phase transition to the 1/2-magnetization plateau phase at 100 T [8, 9], in order to quantitatively estimate the absolute value of the magnetization jump for $LiGaCr_4O_8$ as discussed in the next section. The coaxial-type M pickup coil was employed in this measurement. The temporal evolutions of the magnetic field (gray) and the induction voltage detected by the M pickup coil (blue) at 5 K are shown in Fig. S3(d). As denoted by a triangle in the inset, a sharp dM/dt peak is observed at 100 T, which is consistent with the previous observations obtained in the STC system [8, 9]. Here, additional dM/dt anomalies are observed in a high-field region around 250 T (at 53.2 μs), presumably indicating a phase transition. The interpretation of these observations is out of the scope of this paper.

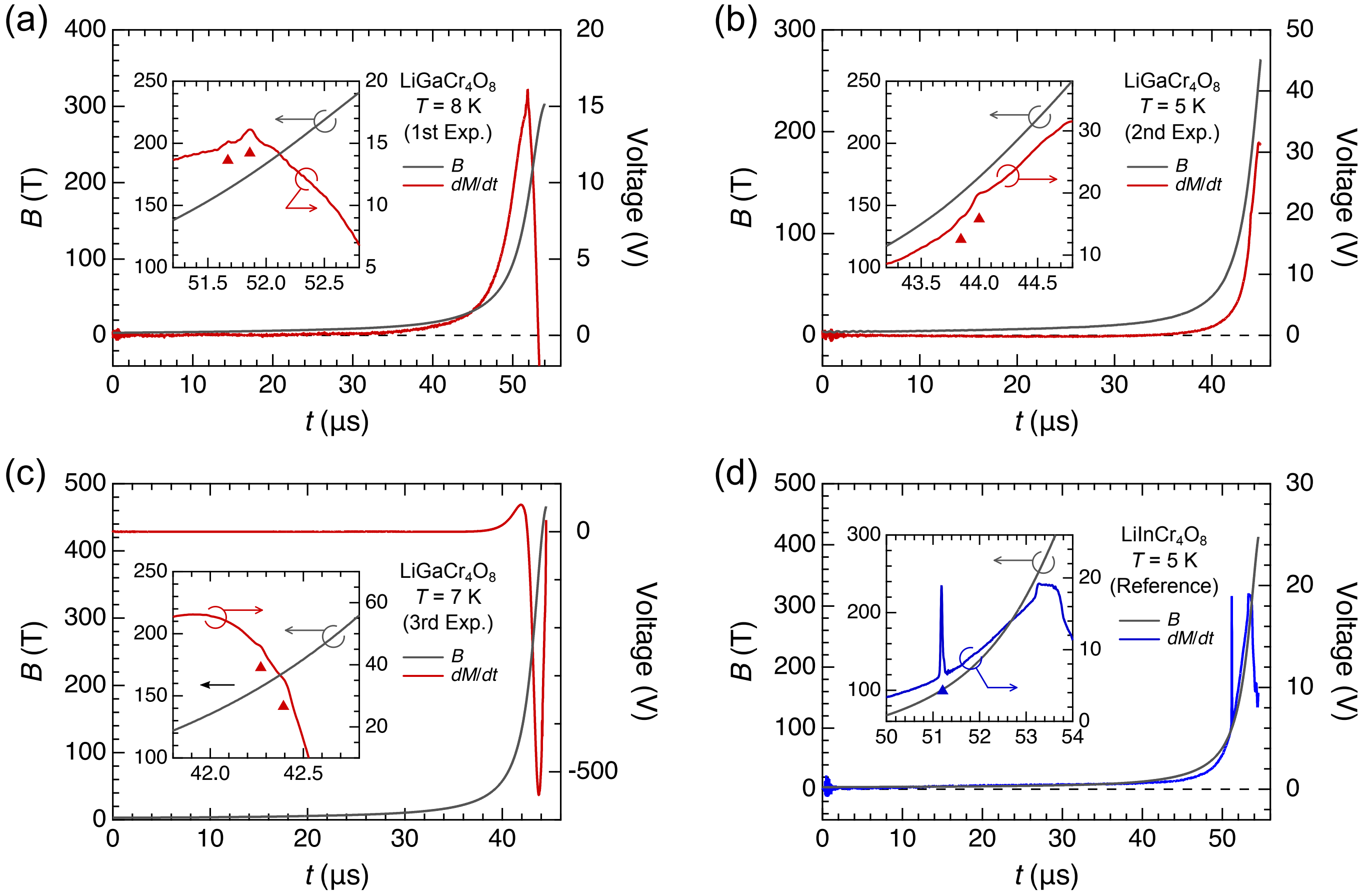

FIG. S6. Temporal evolutions of the magnetic field (gray) and the induction voltage detected by the M pickup coil (red or blue) obtained in the EMFC system. The measurements were performed for $LiGaCr_4O_8$ in (a)–(c) and for $LiInCr_4O_8$ in (d). In all the panels, the inset displays an enlarged view around phase transitions, as denoted by upward triangles in dM/dt data.

Note 5. Analysis of the magnetization data obtained in the EMFC system

We here introduce the procedure to obtain the M–B curve of $LiGaCr_4O_8$ (Fig. 3E) from the raw data (Fig. 3C), which is equivalent to Fig. S6(b).

[Step 1] First, we derive the experimental dM/dB waveform [$(dM/dB)_{exp}$] for $LiGaCr_4O_8$ and $LiInCr_4O_8$ by dividing dM/dt measured by the M pickup coil by dB/dt measured by the B pickup coil. The obtained $(dM/dB)_{exp}$ waveforms for $LiGaCr_4O_8$ and $LiInCr_4O_8$ are shown in Figs. S7(a) and S7(d), respectively.

[Step 2] We approximate a linear background in the $(dM/dB)_{exp}$ waveform in the field region of 150–200 T for $LiGaCr_4O_8$ and 90–110 T for $LiInCr_4O_8$ as shown by the black dashed line in the insets of Figs. S7(a) and S7(d), respectively. Then, we obtain quasi-intrinsic dM/dB waveforms [$(dM/dB)_{sam}$] by subtracting the linear background from the $(dM/dB)_{exp}$ waveforms [Figs. S7(b) and S7(e)].

[Step 3] By integrating $(dM/dB)_{sam}$ as a function of B, M–B curves are obtained as shown in Figs. S7(c) and S7(f). Here, ΔM_{Ga} and ΔM_{In} represent the magnitudes of a magnetization jump accompanied by a phase transition to the 1/2-magnetization plateau. The vertical axes are shown by the same units to enable the quantitative comparison of the experimentally-detected increases in the total magnetic moments. For $LiInCr_4O_8$, the magnitude of the magnetization jump at 100 T is estimated to be $0.6 \pm 0.1\ \mu_B$/Cr by the previous magnetization measurements in the STC system [8, 9]. The corresponding change in Fig. S7(f) is $\Delta M_{In} \approx 93$, while $\Delta M_{Ga} \approx 47$ in Fig. S7(c) as for $LiGaCr_4O_8$. Here, the difference in the number of turns between the inner and outer coils of the employed coaxial M pickup coil was 8 for $LiGaCr_4O_8$ and 11 for $LiInCr_4O_8$. Assuming that the sensitivity of the magnetization detection is proportional to the difference in the number of turns, the magnitude of the magnetization jump in the field region of 150–200 T for $LiGaCr_4O_8$ can be calculated as $(0.6 \pm 0.1) \times (47/93) \times (11/8) = 0.42 \pm 0.07\ \mu_B$/Cr.

[Step 4] We extrapolate the linear M–B curve obtained in the HSTC system as drawn by the black dashed line in Fig. 3E, which can be expressed as $M_{linear}(B)$ [μ_B/Cr] = $0.0048B$ [T]. The final M–B curve in the field region of 150–200 T is derived by adding the M–B curve obtained in Step 3 to the linear component $M_{linear}(B)$.

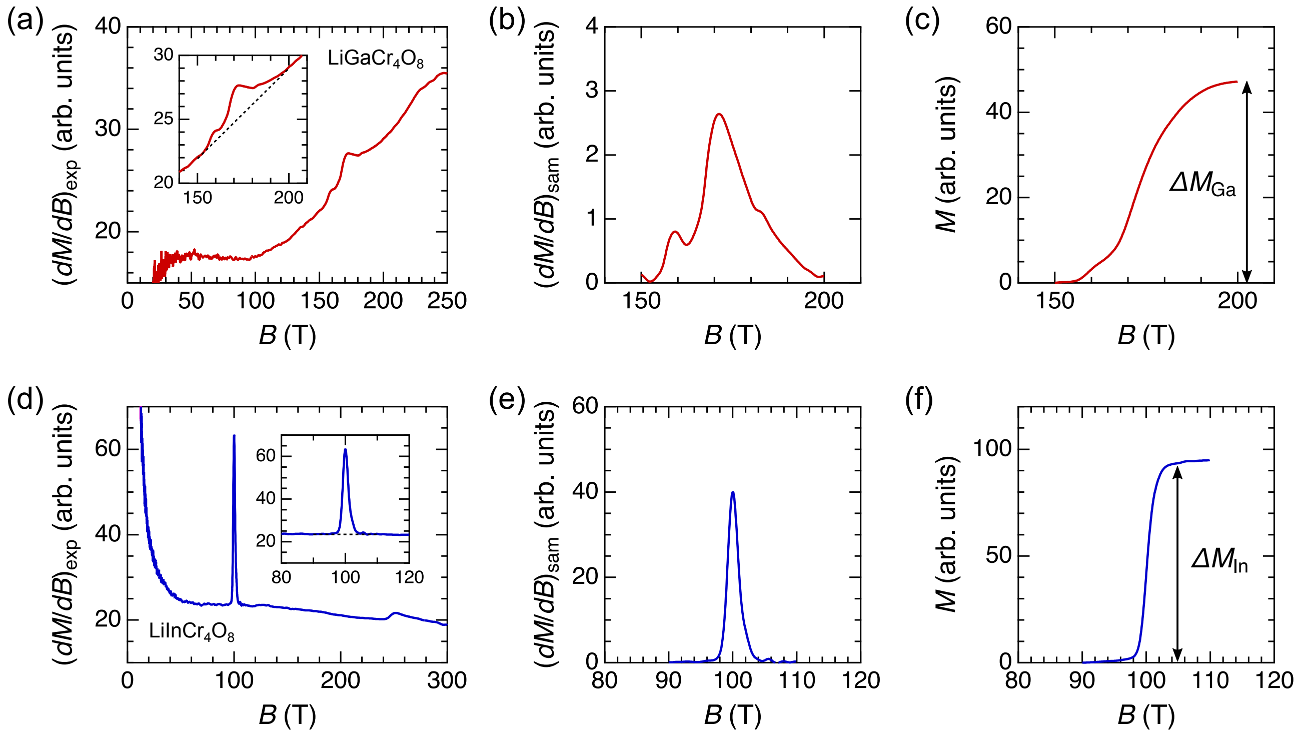

FIG. S7. Procedure for analyzing the magnetization data obtained in the EMFC system for [(a)–(c)] $LiGaCr_4O_8$ and [(d)–(f)] $LiInCr_4O_8$. See the text for details.

Note 6. Details of magnetostriction measurement technique in the EMFC system

For the magnetostriction measurement, we employed the original high-speed strain gauge using fiber-Bragg-grating (FBG) technique and optical filter method implemented in ISSP [13, 14]. In order to efficiently perform the experiment, we have recently extended the FBG measurement system to enable simultaneous measurements of two channels, as schematically shown in Fig. S8(a). Here, C-Band ASE Broadband Light Source (TLA-0016, Amonics Ltd.) is employed. The light source is immediately separated into two branches, and two independent optical filters are applied. The filtered light source illuminates the FBG glued to the sample after passing through an optical circulator. The reflected light from each FBG is guided by the optical circulator and detected with independent InGaAs avalanche photodetectors (APD) (Thorlabs APD430C, conversion gain: 1.8×10^5 V/W, bandwidth: 400 MHz). Finally, the data are acquired using a single oscilloscope (Lecroy HDO4034A, bandwidth: 350 MHz). The development of multi-channel measurements is a major advance in view of the difficulty of performing repetitive experiments with the EMFC system.

In the present work, we performed the FBG experiment twice in the EMFC systems (Shots I and II). In each shot, we placed two $LiGaCr_4O_8$ samples (rod shape with ~0.8 mm in a diameter and ~1.5 mm in a length formed using Stycast 1266) on the probe [Fig. S8(c)] and measured their magnetostriction simultaneously. We show all the data in Supplementary Note 7 and their measurement conditions in Table S2 (shown in the next page). Data #3 and #5 were obtained in Shot I, and Data #4 and #6 in Shot II. We show Data #3 ~ #5 in Fig. 4 in the main text. The sample was located at the center position in the axial direction with respect to the magnet coil for Data #4 and #5, whereas the sample was 10 and 5 mm away from the center in the axial direction for Data #3 and #6, respectively. We also placed two B pickup coils made of AIW wire to measure the magnetic field at each sample position and one RuO_2 tip thermometer.

In the optical filter method, the shift of the Bragg wavelength is converted to the change in the intensity of the optical signal (for details, see Ref. [13]). Here, we used two types of optical filters, (A) 1560SPF and (B) 1551BPF (Koshin Kogaku TFM/FC), both of which allow the tuning of the cut-off wavelength by ±10 nm around $\lambda = 1555$ nm. Transmission spectra of Filters (A) and (B) are displayed in Fig. S8(b). The former allows high sensitivity of $\Delta L/L \sim 1\times10^{-5}$ and a narrow dynamic range of $\Delta L/L \sim 6\times10^{-4}$, whereas the latter allows low sensitivity of $\Delta L/L \sim 5\times10^{-5}$ and a broad dynamic range of $\Delta L/L \sim 3\times10^{-3}$. We used Filter (A) for Data #3 and #6 and (B) for Data #4 and #5. Note that we used Filter (A) to obtain Data #1 and #2 in the STC system (Fig. 3F in the main text).

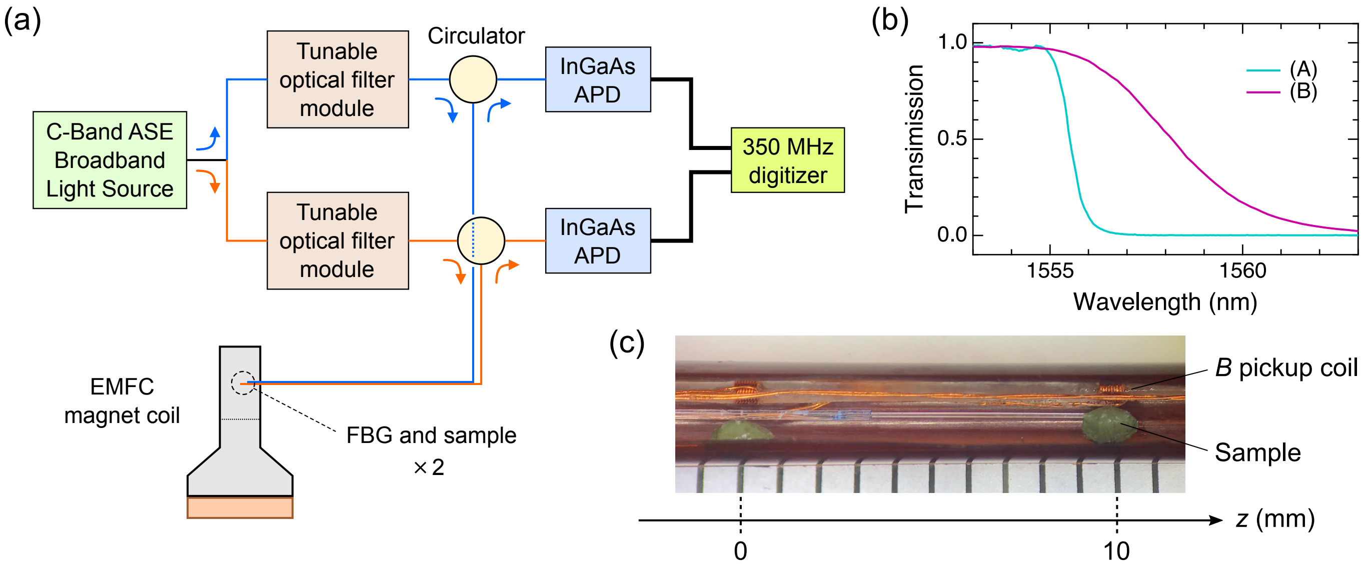

FIG. S8. (a) Block diagram of the renewed high-speed strain monitoring system using the FBG in combination with the optical filter method, enabling simultaneous measurement of two channels. (b) Transmission spectra of the optical filters (A) and (B). (c) Photograph of two FBGs glued to two rod-shaped $LiGaCr_4O_8$ polycrystalline samples placed in a cut-open Kapton tube with ϕ2.5. This setup was used for the experiment of Shot I.

Note 7. All the magnetostriction data obtained in the EMFC system

Figure S9 shows the raw data of the relative sample-length change $\Delta L/L$ obtained by the FBG strain gauge as well as the time dependence of the field strength in the EMFC system. All the measurements were performed at ~5 K. The observed $\Delta L/L$–t curves are significantly affected by oscillation noise above 200 T, which should be caused by the shock wave propagating inside the sample immediately after the ultrafast magnetostructural transitions at B_{c1} and B_{c2} [13]. For Data #3, as the oscillating amplitude remains almost constant between 200 T and B_{max}, no additional phase transition accompanied by an upturn behavior of $\Delta L/L$ is likely to occur. For Data #4 and #5 with $B_{max} \approx 600$ T, on the other hand, a $\Delta L/L$ increase much larger than the negative $\Delta L/L$ behavior caused by the mechanical vibration is reproducibly observed, signaling the occurrence of a phase transition from the 3-up-1-down to a higher-field spin-canted phase.

We note that no oscillation noise is observed in Data #1 and #2 obtained using the STC system, as shown in Figs. 3D and 3F in the main text. This would be because (i) the transition to the 3-up-1-down phase occurs only halfway through due to insufficient B_{max}, and (ii) the transition occurs near B_{max} where the dB/dt becomes small.

TABLE S2. Experimental conditions for all the FBG data obtained in the EMFC system.

Data	Shot	Sample position z	B_{max}	Filter
#3	I	10 mm	360 T	A
#4	II	0 mm	580 T	B
#5	I	0 mm	620 T	B
#6	II	5 mm	440 T	A

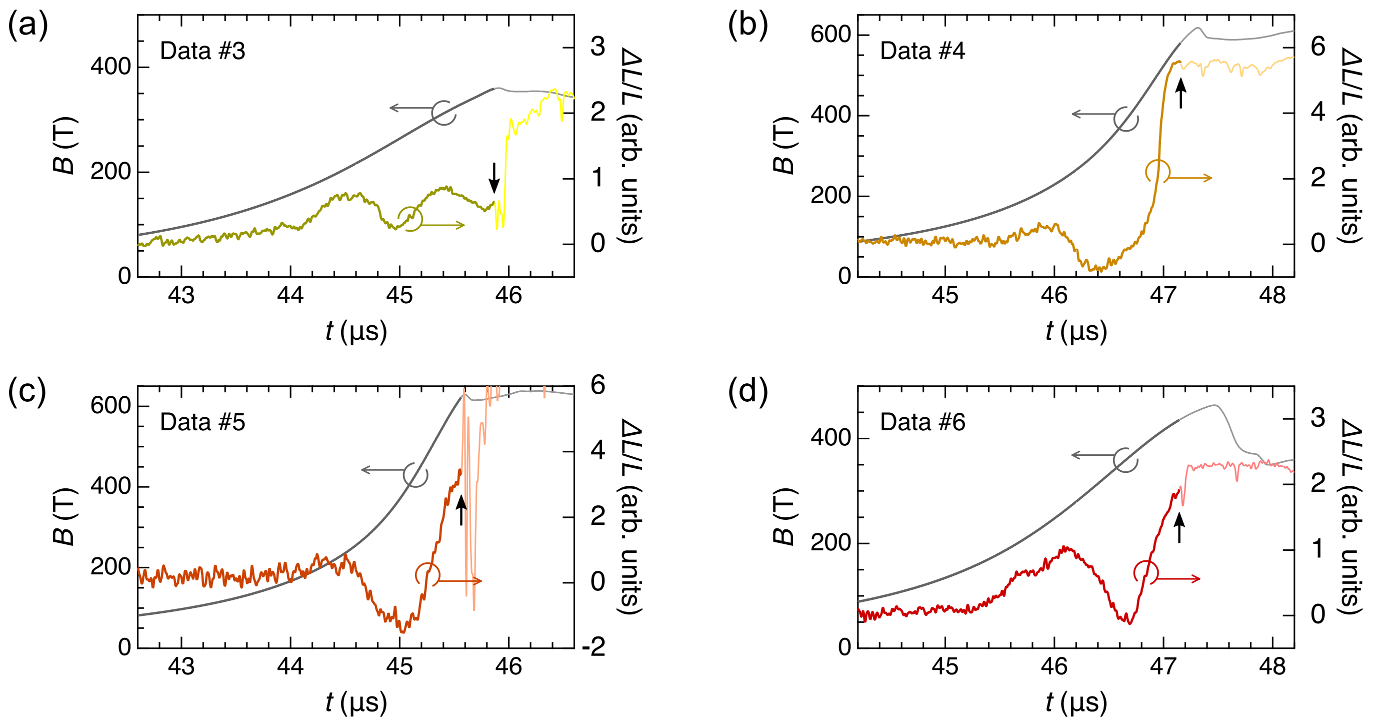

FIG. S9. Temporal evolutions of the magnetic field (left axis) and the relative sample-length change $\Delta L/L$ (right axis) obtained in the EMFC system for (a) Data #3, (b) Data #4, (c) Data #5, and (d) Data #6. The measurement temperature was ~5 K for all data. The thick (thin) lines correspond to the data before (after) the timing of the mechanical breakdown of the probe, which is indicated by black arrows.

Note 8. Analysis of the magnetostriction data obtained in the EMFC system

We present the analysis method to subtract the extrinsic oscillation component from the experimental $\Delta L/L$ data.

We first analyze Data #3 with $B_{\max} = 360$ T. We fit the experimental $[\Delta L/L\,(t)]_{\rm exp}$ curve using the following equation for the damped oscillation

$$[\Delta L/L\,(t)]_{\rm fit} = \alpha + \beta(t - t_0) + Ae^{-\gamma(t-t_0)} \cos\omega(t - t_0), \tag{S1}$$

in a time range of $t_1 \le t \le t_{\max}$, where t_1 and $t_{\max}$ are the time at which the magnetic field reaches 200 T and $B_{\max}$, respectively: $t_1 = 44.448$ [μs], $t_{\max} = 45.868$ [μs]. The obtained fitting curve is shown by a blue line in Fig. S10(a). The values of fitting parameters, α, β, t_0, A, γ, and ω, are summarized in Table S3. The agreement between $[\Delta L/L\,(t)]_{\rm exp}$ and $[\Delta L/L\,(t)]_{\rm fit}$ is good, indicating that the estimation of the oscillation component using the damped oscillation Eq. (S1) is reasonable. Accordingly, we can extract the intrinsic magnetostriction behavior $[\Delta L/L\,(t)]_{\rm int}$ in $t_0 \le t \le t_{\max}$ as shown by a green line in Fig. S10(a) using the following equation

$$[\Delta L/L\,(t)]_{\rm int} = [\Delta L/L\,(t)]_{\rm exp} - A[e^{-\gamma(t-t_0)} \cos\omega(t - t_0) - 1]. \tag{S2}$$

The resultant $\Delta L/L$–B curve is shown in Fig. S10(b).

Based on the above analysis, we analyze Data #4 and #5 with $B_{\max} \sim 600$ T as shown below. We adopt two $\Delta L/L$–B curves shown in Figs. S11(b) and S12(b) in the main text. Note that we avoid detailed analysis for Data #6 because we cannot obtain clear fitting results as well as it is hard to judge whether additional phase transition occurs in a high-field regime.

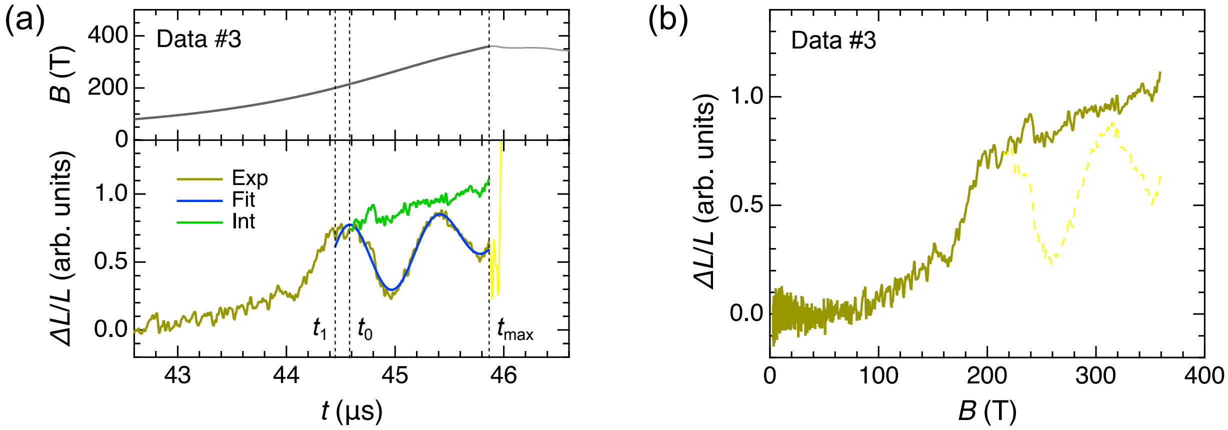

FIG. S10. (a) Temporal evolutions of the magnetic field (upper panel) and the relative sample-length change $\Delta L/L$ (lower panel) obtained in the EMFC system for Data #3. The blue line shows the fitting curve based on Eq. (S1), and the green line shows the estimated intrinsic $\Delta L/L$ component. (b) $\Delta L/L$–B curve before and after subtracting the extrinsic oscillation component for dashed and solid lines.

TABLE S3. List of the fitting parameters in Eq. (S1) adopted to the experimental $\Delta L/L$–t curve in a time rage of $t_1 \le t \le t_2$. Note that $t_2 = t_{\max}$ for Data #3 (see the text for details).

Data	α	β [s^{-1}]	t_0 [μs]	A	γ [s^{-1}]	ω [s^{-1}]
#3	0.40	2.19×10^5	44.584	0.311	4.98×10^5	7.64×10^6
#4	−0.16	0 (fix)	45.937	0.764	4.98×10^5 (fix)	6.73×10^6
#5	−0.37	0 (fix)	44.411	1.065	4.98×10^5 (fix)	5.30×10^6

[Data #4]

Having confirmed that 1/2-magnetization plateau persists from 200 T to at least 360 T from Data #3, we fit the experimental $[\Delta L/L\,(t)]_{\rm exp}$ curve using Eq. (S1) in a time range of $t_1 \le t \le t_2$, where t_2 is the time at which the magnetic field reaches 360 T: $t_1 = 45.804$ [μs], $t_2 = 46.570$ [μs]. Here, we fix $\beta = 0$ because of the difficulty in its estimation and $\gamma = 4.98\times10^5$ [s^{-1}] following the value obtained for Data #6. The obtained fitting curve is shown by a blue line in Fig. S11(a), and the fitting parameters are summarized in Table S3. Finally, we extract the intrinsic magnetostriction behavior $[\Delta L/L\,(t)]_{\rm int}$ in $t_0 \le t \le t_{\rm max}$ as shown by a green line in Fig. S11(a) using Eq. (S2). The resultant $\Delta L/L$–B curve is shown in Fig. S11(b).

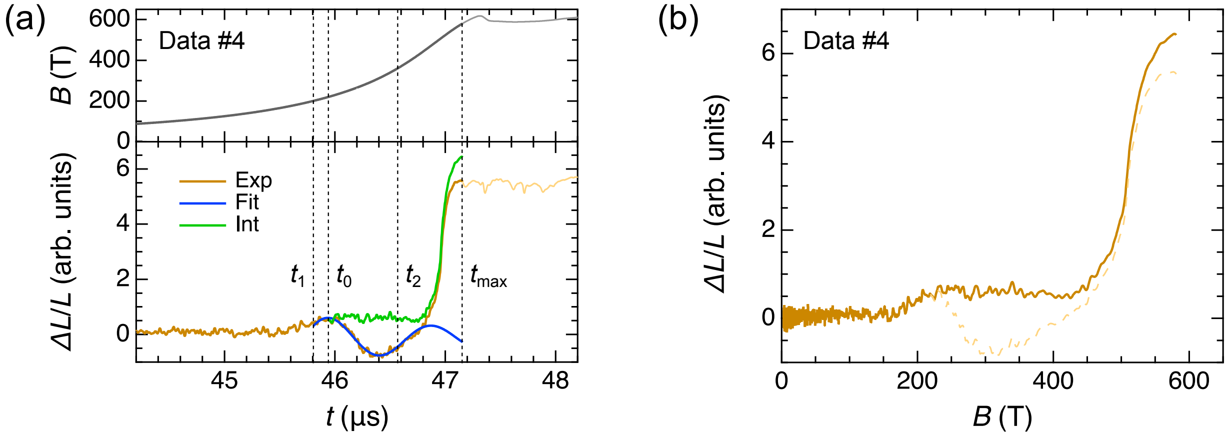

FIG. S11. (a) Temporal evolutions of the magnetic field (upper panel) and the relative sample-length change $\Delta L/L$ (lower panel) obtained in the EMFC system for Data #4 with $B_{\rm max} = 580$ T. The blue line shows the fitting curve based on Eq. (S1), and the green line shows the estimated intrinsic $\Delta L/L$ component. (b) $\Delta L/L$–B curve before and after subtracting the extrinsic oscillation component for dashed and solid lines.

[Data #5]

Similar to the analysis in Data #4, we fit the experimental $[\Delta L/L\,(t)]_{\rm exp}$ curve using Eq. (S1) in a time range of $t_1 \le t \le t_2$: $t_1 = 44.276$ [μs], $t_2 = 44.974$ [μs]. Here, we fix $\beta = 0$ because of the difficulty in its estimation and $\gamma = 4.98\times10^5$ [s^{-1}] following the value obtained for Data #6. The obtained fitting curve is shown by a blue line in Fig. S12(a), and the fitting parameters are summarized in Table S3. Finally, we extract the intrinsic magnetostriction behavior $[\Delta L/L\,(t)]_{\rm int}$ in $t_0 \le t \le t_{\rm max}$ as shown by a green line in Fig. S12(a) using Eq. (S2). The resultant $\Delta L/L$–B curve is shown in Fig. S12(b).

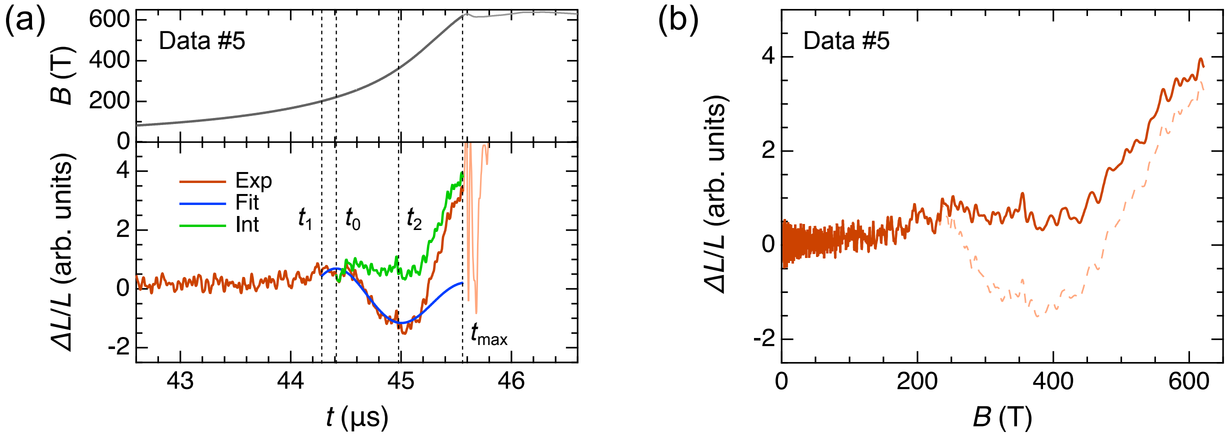

FIG. S12. (a) Temporal evolutions of the magnetic field (upper panel) and the relative sample-length change $\Delta L/L$ (lower panel) obtained in the EMFC system for Data #5 with $B_{\rm max} = 620$ T. The blue line shows the fitting curve based on Eq. (S1), and the green line shows the estimated intrinsic $\Delta L/L$ component. (b) $\Delta L/L$–B curve before and after subtracting the extrinsic oscillation component for dashed and solid lines.

Note 9. Site-phonon model on the breathing pyrochlore Heisenberg antiferromagnet

In order to describe a microscopic magnetoelastic model, we start from the following spin Hamiltonian:

$$\mathcal{H} = \sum_{\langle i,j\rangle} J_{\rm ex}\mathbf{S}_i\cdot\mathbf{S}_j + \frac{c}{2}\sum_i |\mathbf{u}_i|^2 - h\sum_i S_i^z, \tag{S3}$$

where $\langle i,j\rangle$ runs over all the nearest-neighbor (NN) sites, $J_{\rm ex}$ (>0) is the NN AFM exchange interaction, $\mathbf{S}_i$ is the classical spin at site i normalized to $|\mathbf{S}_i| = 1$, c (>0) is the elastic constant, $\mathbf{u}_i$ is the displacement at site i from its original position $\mathbf{r}_i^0$, and h is the strength of the external magnetic field applied along z axis. The exchange striction is introduced assuming $J_{\rm ex}$ linearly modulated by the bond-length change provided that $|\mathbf{u}_i|/|\mathbf{r}_i^0| \ll 1$:

$$J_{\rm ex} \equiv J_{\rm ex}(|\mathbf{r}_{ij}^0 + \mathbf{u}_i - \mathbf{u}_j|) \approx J_{\rm ex}(|\mathbf{r}_{ij}^0|) + \left.\frac{dJ_{\rm ex}}{dr}\right|_{r=|\mathbf{r}_{ij}^0|} \mathbf{e}_{ij}\cdot(\mathbf{u}_i - \mathbf{u}_j), \tag{S4}$$

where $\mathbf{r}_{ij}^0 \equiv \mathbf{r}_i^0 - \mathbf{r}_j^0$ and $\mathbf{e}_{ij} \equiv \mathbf{r}_{ij}^0/|\mathbf{r}_{ij}^0|$. For the lattice degrees of freedom, we assume the so-called Einstein site phonons, where the displacements $\mathbf{u}_i$ are independent of each other [15–17]. Substituting Eq. (S4) to Eq. (S3) and exactly integrating out the lattice degrees of freedom $\mathbf{u}_i$ using the standard Gaussian integration, we obtain

$$\mathcal{H} = \sum_{\langle i,j\rangle} J_{\rm ex}(|\mathbf{r}_{ij}^0|)\mathbf{S}_i\cdot\mathbf{S}_j + \frac{c}{2}\sum_i |\mathbf{u}_i - \bar{\mathbf{u}}_i|^2 - \frac{c}{2}\sum_i |\bar{\mathbf{u}}_i|^2 - h\sum_i S_i^z, \tag{S5}$$

$$\bar{\mathbf{u}}_i = -\frac{1}{c}\sum_{j\in N(i)} \left.\frac{dJ_{\rm ex}}{dr}\right|_{r=|\mathbf{r}_{ij}^0|} \mathbf{e}_{ij}(\mathbf{S}_i\cdot\mathbf{S}_j). \tag{S6}$$

Obviously, the minimization condition is $\mathbf{u}_i = \bar{\mathbf{u}}_i$.

Here, we introduce two kinds of NN exchange interactions $J \equiv J_{\rm ex}(|\mathbf{r}_{ij}^0|_A)$ and $J' \equiv J_{\rm ex}(|\mathbf{r}_{ij}^0|_B)$ within tetrahedra A and B, respectively, in the breathing pyrochlore lattice [see Fig. 2(a) in the main text]. Then, the effective spin Hamiltonian can be expressed as

$$\mathcal{H}_{\rm eff} = \mathcal{H}_0 + \mathcal{H}_{\rm SLC} - h\sum_i S^i \tag{S7}$$

with

$$\mathcal{H}_0 = J\sum_{\langle i,j\rangle_A} \mathbf{S}_i\cdot\mathbf{S}_j + J'\sum_{\langle i,j\rangle_B} \mathbf{S}_i\cdot\mathbf{S}_j, \tag{S8}$$

$$\mathcal{H}_{\rm SLC} = -\frac{c}{2}\sum_i |\bar{\mathbf{u}}_i|^2, \tag{S9}$$

$$\bar{\mathbf{u}}_i = \left\{ \sqrt{\frac{Jb}{c}}\sum_{j\in N_A(i)} + \sqrt{\frac{J'b'}{c}}\sum_{j\in N_B(i)} \right\} \mathbf{e}_{ij}(\mathbf{S}_i\cdot\mathbf{S}_j), \tag{S10}$$

where the dimensionless parameters b (b') represents the strength of the SLC between the NN sites within the tetrahedra A (B), which is defined by $b \equiv (1/cJ)[(dJ/dr)|_{r=|\mathbf{r}_{ij}^0|}]^2$ ($b' \equiv (1/cJ')[(dJ'/dr)|_{r=|\mathbf{r}_{ij}^0|}]^2$), and $N_A(i)$ ($N_B(i)$) denotes a set of the NN sites of site i within tetrahedra A (B). Expanding Eq. (S9), the SLC contribution can be eventually written as

$$\begin{aligned}
\mathcal{H}_{\rm SLC} = &-Jb\sum_{\langle i,j\rangle_A}(\mathbf{S}_i\cdot\mathbf{S}_j)^2 - J'b'\sum_{\langle i,j\rangle_B}(\mathbf{S}_i\cdot\mathbf{S}_j)^2 \\
&-\sum_i \left\{ \frac{Jb}{4}\sum_{j\neq k\in N_A(i)} + \frac{J'b'}{4}\sum_{j\neq k\in N_B(i)} \right\} (\mathbf{S}_i\cdot\mathbf{S}_j)(\mathbf{S}_i\cdot\mathbf{S}_k) \\
&-\sqrt{JJ'bb'}\sum_i\sum_{j\in N_A(i)}\sum_{k\in N_B(i)} \mathbf{e}_{ij}\cdot\mathbf{e}_{ik}(\mathbf{S}_i\cdot\mathbf{S}_j)(\mathbf{S}_i\cdot\mathbf{S}_k),
\end{aligned} \tag{S11}$$

which is identical to Eq. (3) in the main text.

Note 10. System size dependence of the calculated results on Monte Carlo simulations

In our Monte Carlo simulations on the site-phonon model Eq. (S7) with $J'/J = 0.1$, we have checked the system-size $N = 16L^3$ dependence of the calculated results. For $b = b' = 0.05$ and $b = b' = 0.10$, all the calculated results for $L = 4, 6, 8$ and 12 agree with each other. For $b = b' = 0.15$, the energy per site in a field range of $1.4 < h/J < 1.6$ obtained for $L = 6$ and 12 is found to be smaller than that for $L = 4$ and 8 [Fig. S13(a)]. For $L = 6$ and 12, the same $6 \times 6 \times 6$ magnetic long-range order appears with a two-step metamagnetic transition [Fig. S13(b)]. For $L = 4$ and 8, on the other hand, we could not find any signatures of long-range orders with two-fold or four-fold periodicity.

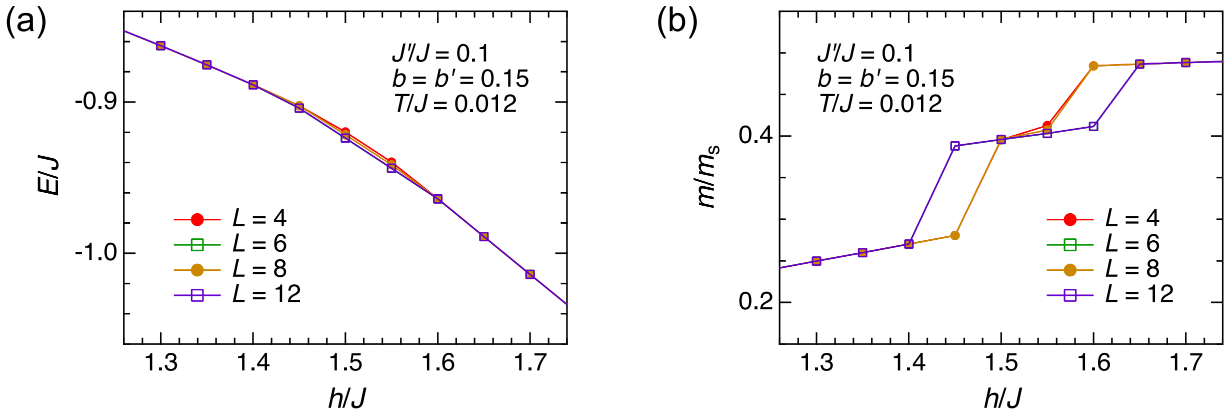

FIG. S13. System size dependence of (a) the energy per site and (b) magnetization in the site-phonon model Eq. (S7) for $J'/J = 0.1, b = b' = 0.015$, and $T/J = 0.012$. The data for $L = 6$ and $L = 12$ perfectly overlap with each other.

Note 11. Magnetic-field width of the half-magnetization plateau

A half-magnetization plateau commonly appears in Cr spinel oxides ACr_2O_4 (A = Zn, Cd, and Hg) [18–20] as well as $LiGaCr_4O_8$. Table S4 summarizes starting and ending fields of the half-magnetization plateau phase in these compounds. Quantitative comparison of the values of B_{c3}/B_{c2} between experiment and theory is useful for discussing the relative field width of the half-magnetization plateau.

TABLE S4. Starting and ending fields of the half-magnetization plateau, B_{c2} and B_{c3}, respectively, in ACr_2O_4 [18–20] and $LiGaCr_4O_8$. All these values were obtained at the lowest measured temperature.

	B_{c2}	B_{c3}	B_{c3}/B_{c2}
$ZnCr_2O_4$	135 T	160 T	1.2
$CdCr_2O_4$	28 T	58 T	2.1
$HgCr_2O_4$	10 T	27 T	2.7
$LiGaCr_4O_8$	~170 T	~420 T	2.5

The ground-state phase diagrams of the bond-phonon and site-phonon models in a regular pyrochlore Heisenberg antiferromagnet ($J'/J = 1, b = b'$) were previously investigated in Ref. [21] and Ref. [17], respectively. Note that, in the bond-phonon model, phonon-mediated spin interactions include only biquadratic terms identical to the first and second terms in Eq. (S11). Figure S14(a) shows the spin-lattice-coupling parameter b dependence of starting and ending fields of the half-magnetization h_{c2} and h_{c3}, respectively, in the aforementioned two kinds of magnetoelastic models with $J'/J = 1$. The increasing rate of h_{c3} with respect to b in the site-phonon model is $dh_{c3}/db = 0.5$, which is exactly half that in the bond-phonon model. The decreasing rate of h_{c2} in the site-phonon model is also exactly half that in the bond-phonon model for $b < 0.05$ and becomes much smaller for $0.05 < b$ (h_{c2} is never less than $0.434 \times 8J$). Consequently, the increase in h_{c3}/h_{c2} with respect to b is slower in the site-phonon model, as shown in Fig. S14(b). As shown in Table S4, B_{c3}/B_{c2} amounts to more than 2 for $CdCr_2O_4$ and $HgCr_2O_4$, which can be reproduced with the bond-phonon model, but not with the site-phonon model. Even with the introduction of breathing anisotropy, i.e., $J'/J \neq 1$, the ground-state phase diagram of the bond-phonon model does not change from that for $J'/J = 1$ given that $b = b'$. For the site-phonon model, h_{c3}/h_{c2} becomes a bit larger than the case of $J'/J = 1$ but never exceeds 2 unlike the bond-phonon model: $h_{c3}/h_{c2} \approx 1.23$ for $b = b' = 0.05$, $h_{c3}/h_{c2} \approx 1.56$ for $b = b' = 0.10$, and $h_{c3}/h_{c2} \approx 1.63$ for $b = b' = 0.15$ (Figs. 5A–5C in the main text). From the above, we conclude that the site-phonon model underestimates the field width of the half-magnetization plateau.

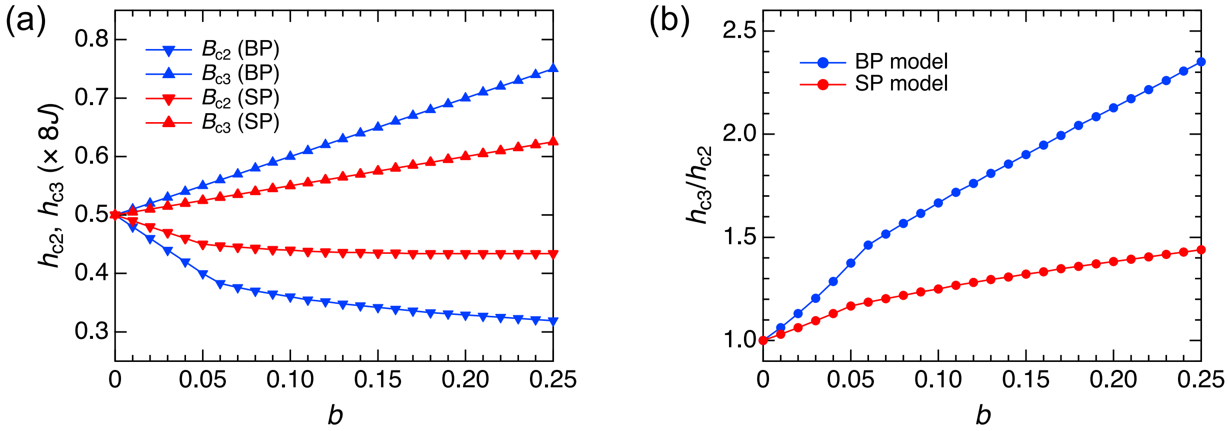

FIG. S14. Spin-lattice-coupling parameter ($b = b'$) dependence of (a) starting and ending fields of the half-magnetization plateau, h_{c2} (downward triangles) and h_{c3} (upward triangles), respectively, and (b) the value of h_{c3}/h_{c2} in the bond-phonon (BP, blue) and site-phonon (SP, red) models with $J'/J = 1$.

* masaki.gen@riken.jp
† ykohama@issp.u-tokyo.ac.jp

[1] Y. Okamoto, G. J. Nilsen, J. P. Attfield, and Z. Hiroi, Breathing Pyrochlore Lattice Realized in *A*-Site Ordered Spinel Oxides $LiGaCr_4O_8$ and $LiInCr_4O_8$, Phys. Rev. Lett. **110**, 097203 (2013).
[2] R. Saha, F. Fauth, M. Avdeev, P. Kayser, B. J. Kennedy, and A. Sundaresan, Magnetodielectric effects in *A*-site cation-ordered chromate spinels $LiMCr_4O_8$ (M = Ga and In), Phys. Rev. B **94**, 064420 (2016).
[3] S. Takeyama, K. Amaya, T. Nakagawa, M. Ishizuka, T. Sakakibara, T. Goto, N. Miura, Y. Ajiro, and H. Kikuchi, Magnetisation measurements in ultra-high magnetic fields produced by a single-turn coil system, J. Phys. E **21**, 1025 (1988).
[4] K. Amaya, S. Takeyama, T. Nakagawa, M. Ishizuka, K. Nakao, T. Sakakibara, T. Goto, N. Miura, Y. Ajiro, and H. Kikuchi, Magnetization measurements in very high plused fields produced by a single-turn coil system, Physica B **155**, 396 (1989).
[5] T. Goto, H. Aruga Katori, T. Sakakibara, and M. Yamaguchi, Successive phase transitions in ferromagnetic YCo_3, Physica B **177**, 255 (1992).
[6] A. Kirste, M. Goiran, M. Respaud, J. Vanaken, J. M. Broto, H. Rakoto, M. von Ortenberg, and J. L. Garcia-Munoz, High magnetic field study of charge melting in $Bi_{1/2}(Sr,Ca)_{1/2}MnO_3$ perovskites: Unconventional behavior of bismuth charge ordered compounds, Phys. Rev. B **67**, 134413 (2003).
[7] S. Takeyama, R. Sakakura, Y. H. Matsuda, A. Miyata, and M. Tokunaga, Precise Magnetization Measurements by Parallel Self-Compensated Induction Coils in a Vertical Single-Turn Coil up to 103 T, J. Phys. Soc. Jpn. **81**, 014702 (2012).
[8] Y. Okamoto, D. Nakamura, A. Miyake, S. Takeyama, M. Tokunaga, A. Matsuo, K. Kindo, and Z. Hiroi, Magnetic transitions under ultrahigh magnetic fields of up to 130 T in the breathing pyrochlore antiferromagnet $LiInCr_4O_8$, Phys. Rev. B **95**, 134438 (2017).
[9] M. Gen, D. Nakamura, Y. Okamoto, and S. Takeyama, Ultra-high magnetic field magnetic phases up to 130 T in a breathing pyrochlore antiferromagnet $LiInCr_4O_8$, J. Magn. Magn. Mater. **473**, 387 (2019).
[10] M. Gen, Y. Okamoto, M. Mori, K. Takenaka, and Y. Kohama, Magnetization process of the breathing pyrochlore magnet $CuInCr_4S_8$ in ultrahigh magnetic fields up to 150 T, Phys. Rev. B **101**, 054434 (2020).
[11] D. Nakamura, S. Sawabe, and S. Takeyama, Note: Experimental evidence of three-dimensional dynamics of an electromagnetically imploded liner, Rev. Sci. Instrum. **85**, 036102 (2014).
[12] D. Nakamura, A. Ikeda, H. Sawabe, Y. H. Matsuda, and S. Takeyama, Record indoor magnetic field of 1200 T generated by electromagnetic flux-compression, Rev. Sci. Instrum. **89**, 095106 (2018).
[13] A. Ikeda, T. Nomura, Y. H. Matsuda, S. Tani, Y. Kobayashi, H. Watanabe, and K. Sato, High-speed 100 MHz strain monitor using fiber Bragg grating and optical filter for magnetostriction measurements under ultrahigh magnetic fields, Rev. Sci. Instrum. **88**, 083906 (2017).
[14] A. Ikeda, Y. H. Matsuda, K. Sato, Y. Ishii, H. Sawabe, D. Nakamura, S. Takeyama, and J. Nasu, Signature of spin-triplet exciton condensations in $LaCoO_3$ at ultrahigh magnetic fields up to 600 T, Nat. Commun. **14**, 1744 (2023).
[15] D. L. Bergman, R. Shindou, G. A. Fiete, and L. Balents, Models of degeneracy breaking in pyrochlore antiferromagnets, Phys. Rev. B **74**, 134409 (2006).
[16] K. Aoyama and H. Kawamura, Spin ordering induced by lattice distortions in classical Heisenberg antiferromagnets on the breathing pyrochlore lattice, Phys. Rev. B **99**, 144406 (2019).
[17] K. Aoyama, M. Gen, and H. Kawamura, Effects of spin-lattice coupling and a magnetic field in classical Heisenberg antiferromagnets on the breathing pyrochlore lattice, Phys. Rev. B **104**, 184411 (2021).
[18] S. Kimura, M. Hagiwara, T. Takeuchi, H. Yamaguchi, H. Ueda, Y. Ueda, and K. Kindo, Large change in the exchange interactions of $HgCr_2O_4$ under very high magnetic fields, Phys. Rev. B **83**, 214401 (2011).
[19] A. Miyata, S. Takeyama, and H. Ueda, Magnetic superfluid state in the frustrated spinel oxide $CdCr_2O_4$ revealed by ultrahigh magnetic fields, phys. Rev. B **87**, 214424 (2013).
[20] A. Miyata, H. Ueda, Y. Ueda, H. Sawabe, and S. Takeyama, Magnetic Phases of a Highly Frustrated Magnet, $ZnCr_2O_4$, up to an Ultrahigh Magnetic Field of 600 T, Phys. Rev. Lett. **107**, 207203 (2011).
[21] K. Penc, N. Shannon, and H. Shiba, Half-Magnetization Plateau Stabilized by Structural Distortion in the Antiferromagnetic Heisenberg Model on a Pyrochlore Lattice, Phys. Rev. Lett. **93**, 197203 (2004).
